# A Smartphone-Based Health Care Chatbot to Promote Self-Management of Chronic Pain (SELMA): Pilot Randomized Controlled Trial

**DOI:** 10.2196/15806

**Published:** 2020-04-03

**Authors:** Sandra Hauser-Ulrich, Hansjörg Künzli, Danielle Meier-Peterhans, Tobias Kowatsch

**Affiliations:** 1 Department of Applied Psychology University of Applied Sciences Zurich Zurich Switzerland; 2 Department Primary School Division Education, Culture and Sports Aarau Switzerland; 3 Center for Digital Health Interventions Institute of Technology Management University of St Gallen St Gallen Switzerland; 4 Center for Digital Health Interventions Department of Management, Technology, and Economics ETH Zurich Zurich Switzerland

**Keywords:** conversational agent, chatbot, digital health, pain self-management, cognitive behavior therapy, smartphone, psychoeducation, text-based, health care, chronic pain

## Abstract

**Background:**

Ongoing pain is one of the most common diseases and has major physical, psychological, social, and economic impacts. A mobile health intervention utilizing a fully automated text-based health care chatbot (TBHC) may offer an innovative way not only to deliver coping strategies and psychoeducation for pain management but also to build a working alliance between a participant and the TBHC.

**Objective:**

The objectives of this study are twofold: (1) to describe the design and implementation to promote the chatbot painSELfMAnagement (SELMA), a 2-month smartphone-based cognitive behavior therapy (CBT) TBHC intervention for pain self-management in patients with ongoing or cyclic pain, and (2) to present findings from a pilot randomized controlled trial, in which effectiveness, influence of intention to change behavior, pain duration, working alliance, acceptance, and adherence were evaluated.

**Methods:**

Participants were recruited online and in collaboration with pain experts, and were randomized to interact with SELMA for 8 weeks either every day or every other day concerning CBT-based pain management (n=59), or weekly concerning content not related to pain management (n=43). Pain-related impairment (primary outcome), general well-being, pain intensity, and the bond scale of working alliance were measured at baseline and postintervention. Intention to change behavior and pain duration were measured at baseline only, and acceptance postintervention was assessed via self-reporting instruments. Adherence was assessed via usage data.

**Results:**

From May 2018 to August 2018, 311 adults downloaded the SELMA app, 102 of whom consented to participate and met the inclusion criteria. The average age of the women (88/102, 86.4%) and men (14/102, 13.6%) participating was 43.7 (SD 12.7) years. Baseline group comparison did not differ with respect to any demographic or clinical variable. The intervention group reported no significant change in pain-related impairment (*P*=.68) compared to the control group postintervention. The intention to change behavior was positively related to pain-related impairment (*P*=.01) and pain intensity (*P*=.01). Working alliance with the TBHC SELMA was comparable to that obtained in guided internet therapies with human coaches. Participants enjoyed using the app, perceiving it as useful and easy to use. Participants of the intervention group replied with an average answer ratio of 0.71 (SD 0.20) to 200 (SD 58.45) conversations initiated by SELMA. Participants’ comments revealed an appreciation of the empathic and responsible interaction with the TBHC SELMA. A main criticism was that there was no option to enter free text for the patients’ own comments.

**Conclusions:**

SELMA is feasible, as revealed mainly by positive feedback and valuable suggestions for future revisions. For example, the participants’ intention to change behavior or a more homogenous sample (eg, with a specific type of chronic pain) should be considered in further tailoring of SELMA.

**Trial Registration:**

German Clinical Trials Register DRKS00017147; https://tinyurl.com/vx6n6sx, Swiss National Clinical Trial Portal: SNCTP000002712; https://www.kofam.ch/de/studienportal/suche/70582/studie/46326.

## Introduction

Chronic pain is a widespread medical condition associated with significant negative social, physical, mental, and economic impacts [1]. The prevalence of chronic pain in Europe is estimated between 10% and 30% [2] and approximately 30% for the US population [3]. In terms of economic impact, costs associated with chronic pain in Germany were estimated to reach 29 billion EUR in 2003, with absence from work due to pain estimated at a total of 14.5 million days per year [4].

Current psychological approaches to the management of chronic pain include interventions that aim to achieve increased self-management, behavior change, and cognitive change rather than cure of the pain itself [5]. Cognitive behavioral therapy (CBT) has proven to be effective and is considered the standard therapy in chronic pain treatment [6-9]. The main focus of CBT is to establish new or enhance existing coping strategies in pain management to change negative and dysfunctional cognitions, emotions, and behavior [5,10]. As part of CBT, psychoeducation shows positive therapeutic effects and can effectively counter the process of chronification [11,12]. Psychoeducation increases patients’ understanding of their condition, thereby improving compliance and helping patients to better cope with the disease.

Despite agreement on a multimodal therapy approach, according to a World Health Organization (WHO) study [13], CBT is typically not sufficiently applied and many patients do not receive CBT as part of multimodal therapy. These gaps are mainly attributed to the lack of therapists’ familiarity with elements of CBT, the lack of qualified providers, or that the only available providers are located too far away for on-site consultation hours. Further barriers include lack of time, insufficient financial resources, and misunderstanding of patients’ roles in their pain management [14]. Research has shown that some patients are not successful in learning new coping strategies [15,16] and remain skeptical about implementing psychological strategies.

Technology-based interventions for the management of chronic pain are becoming more popular [7,14,17], offering a potential solution to overcome these barriers to treatment because they are easily accessible and cost-effective. Further advantages of technology-based interventions include less waiting time, anonymity, and flexibility in terms of time and location of use [18,19]. These interventions are ubiquitous, with increasingly powerful technical abilities and wide acceptability [20].

In this context, so-called conversational agents (ie, computer programs that imitate communication with humans) with a health focus have recently gained interest in academia and industry, with promising results related to acceptance [21] and working alliance [22]. Working alliance refers to the extent to which a therapist and client build an attachment bond and interact with each other to achieve a shared understanding of therapeutic goals and tasks [23], which has been robustly linked to treatment success in both face-to-face and guided web-based programs [24-27]. However, computers, provided that they offer basic human cues (eg, speech output or a human-like embodiment), are perceived as social actors [28]. The concept of working alliance can be adopted to the patterns of interaction between the participant and conversational agent (eg, quality and length of messages, frequency of interaction). If the conversational agent takes the role of a communication partner and embodies a digital coach, the communication style will affect the process of relationship building and hence treatment success [22,29]. Indeed, evidence from longitudinal studies suggests that a working alliance can also be established with conversational agents [22,30]. With the help of conversational agents, interventions can be applied in a more natural and interactive way [31,32]. Digital health interventions delivered via text messages on smartphones have the potential to support patients in their everyday life, as they are cheap, fast, democratic, and popular [33]. A recent meta-analysis showed the effectiveness of conversational agents in 19 clinical and nonclinical randomized controlled trials [34].

However, there is only limited evidence on chronic pain management interventions delivered by smartphone-based conversational agents. Shamekhi et al [35] demonstrated that a home-based conversational agent can be effective when used in conjunction with medical group visits in promoting stress management techniques. However, to the best of our knowledge, no study has investigated the effectiveness of a smartphone-based conversational agent as a stand-alone intervention.

Consequently, we developed painSELfMAnagement (SELMA) as a text-based health care chatbot (TBHC) for the self-management of chronic pain. A TBHC is a conversational agent that supports health professionals in the delivery of evidence-based interventions in a ubiquitous and fully automated fashion with simple text-based messages and media objects (eg, videos, podcasts). A TBHC aims to deliver the treatment and to increase working alliance by communicating therapeutic goals and tasks in an empathetic way [36,37].

Against this background, we here describe the design and implementation of SELMA, an 8-week smartphone-based TBHC intervention for self-management of pain by patients with ongoing or cyclic pain, and present findings from a pilot randomized controlled trial, in which effectiveness, acceptance, and adherence were evaluated.

## Methods

### Intervention

#### Overview

**Figure 1:**
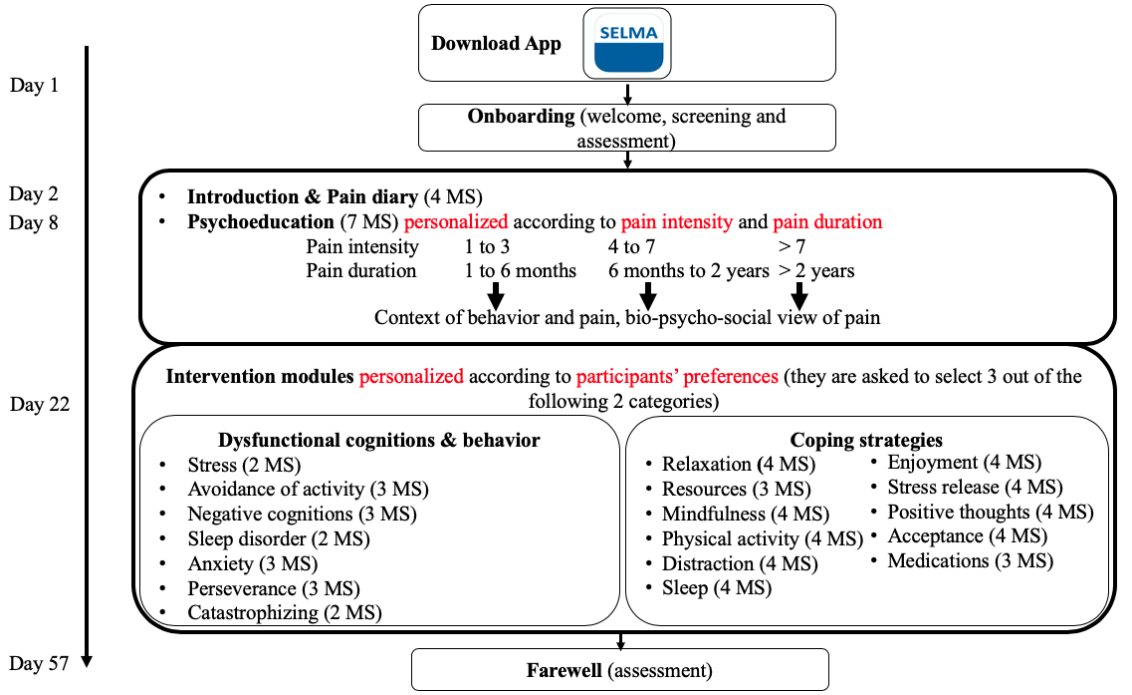
Overview of the intervention schedule. Note: One message sequence (MS) contains approximately 15 conversational turns and takes about 5 to 20 minutes to complete, depending on whether or not an exercise is involved.

The pain self-management coach is implemented as a TBHC named SELMA, which is represented by a drawn image of a face and acts as a guide through the CBT lesson materials. SELMA is displayed via text messages, and complemented introductions and elaborations about the material based on previous answer options and themes of particular interest to the participant. First, SELMA delivers psychoeducation from day 1 to 21 in 7 daily or other daily text message sequences that follow the same structure: initial greeting, psychoeducational input, main lesson, and goodbye. Psychoeducation enhances peoples’ understanding of their disease while reducing insecurity and feelings of fear. This approach further creates transparency and reduces stereotypes against a biopsychosocial pain therapy, while strengthening therapy motivation and self-help potential [38]. From the period of day 22 until the end, SELMA delivers CBT intervention modules addressing coping strategies, along with dysfunctional cognitions and emotions that follow the same structure: initial greeting, introduction, main lesson, guidance through an exercise, goodbye. Coping strategies empower participants to take over an active role in their pain management, enabling them to control pain by analyzing and adapting their behavior, emotions, and cognitions [39]. Depending on whether or not a message sequence involves an exercise, it takes about 5 to 30 minutes to complete. Each of the 18 intervention modules consists of 2 to 4 message sequences (see Figure 1).

#### Content Design and Schedule

The program starts with the onboarding process, an introductory process in which the TBHC SELMA introduces herself as a digital coach for pain self-management. SELMA provides an overview of the intervention schedule, mentions the importance of applying psychological therapy, and the importance of diagnostics in pain management. Participants are also informed that the program should not be used as a replacement for standard therapy and urges users to make an emergency call if necessary. During the first week, participants learn about the utility of a pain diary and are instructed to apply it over the following 2 weeks. In parallel, and until the end of week 3, SELMA provides information on psychoeducation based on the participants’ input with regard to pain intensity and duration. SELMA explains the link between chronic pain, cognition, emotions, social impacts, and the process of chronification. During weeks 4 to 8, SELMA offers various intervention modules. Based on the participants’ interest, they are able to select among 6 modules that either address dysfunctional cognitions, behavior, and emotions (eg, stress, fear of pain, anxiety) or various coping strategies (eg, activity, resources, mindfulness, acceptance). For example, the module about dysfunctional behavior covers *avoidance of activity*. It explains the link between avoiding activity and pain, and SELMA motivates participants to start physical activity and reminds them to keep their own level of proficiency in mind. By contrast, the module *mindfulness*, a coping strategy module, briefly explains the concept of mindfulness and provides users with a mindfulness exercise. Specifically, SELMA instructs participants on how to integrate mindfulness into their daily routine and provides users with a relaxation exercise. Figure 1 shows an overview of the intervention schedule, and Multimedia Appendix 1 provides an overview of the weekly core themes and tasks to complete (including 19 references [10,38-55]). The program closes with a brief summary and farewell. All content is delivered via text messages, video clips, audio clips, figures, and PDF worksheets.

#### Technical Concept

Technically, SELMA was developed with MobileCoach [56], an open-source software platform for the design and evaluation of mobile TBHCs [31,55]. This platform allows the intervention authors to design (fully automated and script-based) digital health interventions consistent with the talk-and-tools paradigm [57]. That is, SELMA offers a simple chat-based interface with predefined answer options that can be used to communicate with participants in (the “talk”). To build up a social relationship [28] and working alliance [22] with participants, the linguistic style of SELMA was based on the assumption that interpersonal closeness is positively related to the attachment bond between the patient and chatbot [25,58,59]. The conversational style of SELMA is likely to affect relationship-building processes [30,60], and imitates a real human chat-based conversation by using emojis and some sense of humor. Supportive computer agents have been perceived positively [61-63]. SELMA expresses sympathy and affective empathy [64], and places emphasis on the participants’ achieved tasks. This approach is based on social support [65,66] and aims for a supportive style of coaching. To engage participants further, SELMA sends out personalized text messages every day or every other day to initiate a conversation.

#### Message Structure and Reminders

Daily conversations are structured as a sequence of approximately 10 to 15 messages sent by SELMA, and approximately 5 to 10 replies are expected from the participants. SELMA is always initiating a conversational turn. Every text message sent by SELMA is also a notification. If the SELMA app is closed and SELMA sends a message, then a notification will always be triggered. These notifications are sticky, meaning that they are displayed in the notification dashboard and thus act as reminders. If the app is already opened, then no additional notification is triggered. To reduce the burden of the intervention, no additional reminders were used (eg, text message). In addition, if the app is closed and the push notifications are not clicked or if the app is not running in the foreground but no answer is provided (usually for 48 hours), then SELMA starts with the next message sequence. Even if participants forget to open the app or read messages (not clicking the push notification or not clicking answer options), SELMA displays the missed messages, together with the next message sequence. This mechanism ensures that participants can read all messages from SELMA, including missed messages, by simply scrolling back. Moreover, this approach is consistent with prior work [37,67,68]. To ensure completion of the questionnaire, every question displayed on one screen had to be completed; otherwise, participants were unable to proceed. Participants were excluded from the analyses if questionnaires were not completed.

#### Implementation

Each message sequence begins with a warm greeting, in which the chatbot enquires about the participant’s mood and replies in an empathic way (eg, “Welcome back dear [$nickname]. It’s nice to have you with me today.”) [69]. Multimedia Appendix 2 shows examples of the sympathetic and affective empathy elements of conversation. To support establishment of a working alliance, user engagement, and motivation, SELMA addresses participants’ accountability by referring to earlier tasks and activities (eg, “Welcome back to the coaching! Were you able to relax yesterday?”); she supports the completion of activities and tasks (eg, “Hi [$nickname]. How is it going with practicing your exercises?”), and motivates participants to repeat them (eg, “How did you manage the exercise, perhaps you can repeat it before the next time we meet?”).

**Figure 2:**
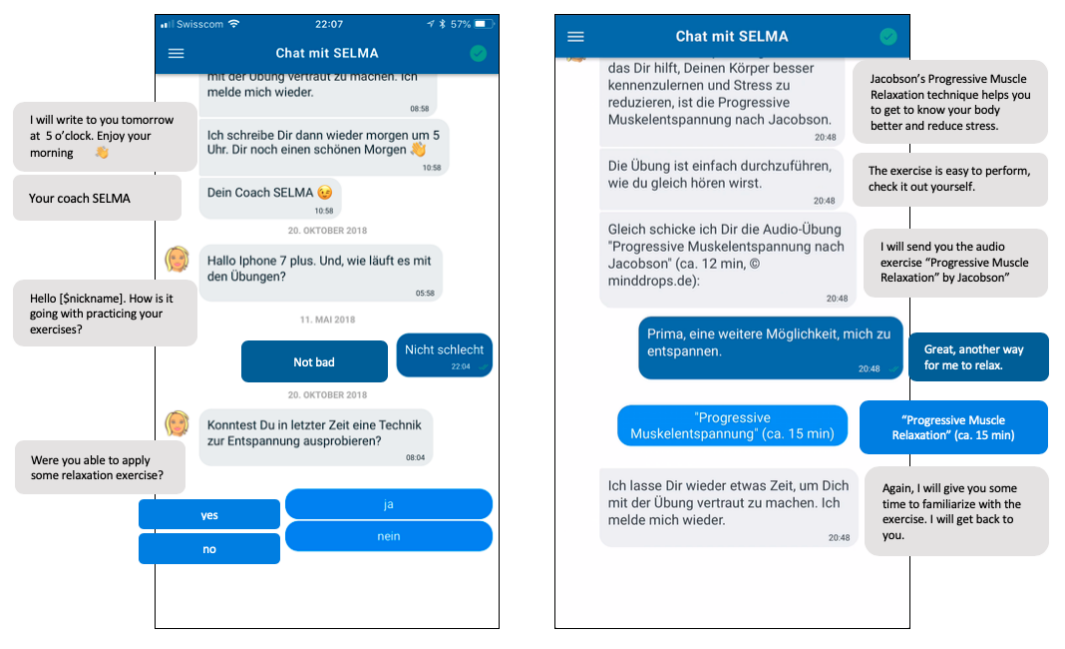
Chat-based interaction with predefined answer options (bright blue, left) and multimedia content (bright blue, right); for further examples, see Multimedia Appendix 3.

In addition to this chat-based messaging interface, “tools” are provided that support the delivery of the intervention content. For example, SELMA offers audio clips or video clips from within the chat-based interface. Figure 2 provides a representative example of an interaction with SELMA and further examples are shown in Multimedia Appendix 3.

Based on intervention rules, SELMA sends out automated messages containing intervention content or questions to the mobile (iOS or Android) apps of the participants. Based on answers given to predefined answer options (eg, from a multiple-choice question on coping strategies), free text input (eg, a question asking for the participant’s nickname), or number input fields (eg, a question asking the participant’s age), participants are guided through the program as outlined in Multimedia Appendix 1 and Figure 1. Some screening questions are embedded as specific focal points to determine routing to different conversational paths. The path parameters therefore change over the course of the program, depending on the user’s input about prior knowledge and interest (pain intensity, pain duration, and preferred intervention modules); see Table 1 for an overview. Predefined answer options were predominantly used to assure reproducibility of the intervention. That is, automated interpretation of free-text input would result in uncertainty by triggering follow-up intervention content. From an ethical viewpoint, this was not intended. To ensure the continuance of the program and to guide those who do not want to actively choose a module (coping strategies or dysfunctional cognitions are focal points), the system displays default modules. SELMA informs participants when a module is finished, and the following day or the day after, she sends out an overview on completed modules and displays all modules for selection. The video clip in Multimedia Appendix 4 demonstrates a sample conversation.

**Table 1:** Information gathered during the intervention.

Information gathered		Input option
**Onboarding**		
	Name	Free text input
	Gender	Male/female
	Age	Free number input
	Time of contact	5.00-20.00 h, every full hour
**Day 1 to 20**		
	Pain intensity (1 to 10)	1 to 3, 4 to 7, more than 7 (3 groups)
	Pain duration (2 months to >5 years)	Up to 2 years, more than 2 years (2 groups)
**Day 20 to 57**		
	Interest in dysfunctional cognitions	Choice of 7 modules (to choose a total of 3)
	Interest in coping strategies	Choice of 11 modules (to choose a total of 3)
**Day 57**		
	Appealing aspects	Free-text input
	Aspects for improvement	Free-text input

### Assessment of the SELMA Intervention

We conducted a randomized controlled pilot trial to assess the SELMA intervention. The clinical trial was approved by the Cantonal Ethics Committee of Zurich (KEK-ZH study protocol identifier Nr. 2017-02136) and was registered at the Swiss National Clinical Trial Portal (SNCTP000002712) and the WHO-accredited German Clinical Trials Register (DRKS00017147). Data protection requirements were fulfilled according to the KEK-ZH.

### Sample Size Calculation

We estimated the sample size based on the primary outcome (pain-related impairment) for a linear mixed model (LMM) and on the basis of a repeated-measures analysis of variance (ANOVA). Consistent with previous studies [70-72], we assumed a small to medium effect size for the primary outcome, pain-related impairment. Statistical power calculation using G*Power^3^ software [73] revealed that a sample size of 68 would be sufficient with a power of .80 to detect a small to medium time-by-group interaction effect (Cohen *d*=0.35) for pain-related impairment with an alpha of .05 by applying a repeated-measures ANOVA (within-between interaction). To ensure a sufficient number of participants, we aimed for approximately 115 individuals with ongoing or cyclic pain.

### Recruitment

Recruitment was carried out from March 2018 to August 2018 in the German-speaking part of Switzerland. We recruited potential participants by flyers posted on social media websites (Facebook, Twitter), pain-related websites and forums (SchmerzLOS e.V. [74], MyHandicap [75], Paincompanion [76]) or via the project webpage (Multimedia Appendix 5). We further asked some pain therapists, physiotherapists, and osteopathy therapists in the agglomeration of Zug (Switzerland) to publish a flyer in their waiting rooms (Multimedia Appendix 6). Interested individuals were directed to the project website containing information about the study, participation, and registration. During the subject acquisition phase, individuals were able to download the app via the project webpage or directly via App Store or Google Play Store.

### Inclusion Criteria, Informed Consent, and Intervention Process

Because of the exploratory nature of this study and to reach a sufficient sample size, we did not focus on a particular type of pain but rather included anyone suffering from ongoing pain. Inclusion criteria were checked from within the mobile SELMA app after the download and included: age (>18 years), language (German-speaking), owning a smartphone with internet access, pain duration (a minimum of 2 months of ongoing or cyclic pain), and not suffering from an acute mental crisis. Electronic informed consent was obtained from all participants who fulfilled the inclusion criteria (T0 screening), and these confirmed individuals were then guided to the baseline screening (T1). All screenings were carried out in the app’s inbuilt assessment questionnaire via LimeSurvey (V3.4, LimeSurvey Project, Hamburg, Germany). After completion of the screenings, participants were automatically randomized by the MobileCoach system to either the intervention or control group. After the 8-week intervention, the follow-up screening (T2) was conducted with the app’s inbuilt questionnaire. Individuals from the control group were informed of their group allocation and were offered participation in the program after the waiting time. These users received motivational messages with a quotation every week, which only involved content unrelated to chronic pain (Multimedia Appendix 7). The postintervention screening was conducted after the 8-week waiting time within the app.

### Measures

#### Primary Outcome: Pain-Related Impairment

An overview of the measures and time points of measurement are outlined in Multimedia Appendix 8.

We measured the primary outcome at baseline and postintervention using a 7-item subscale of the German version of the validated Brief Pain Inventory (BPI) [77]. On a numeric scale from 0 (no impairment at all) to 10 (greatest impairment), impairments in a person’s everyday life (eg, activity, work, relations) are quantified. We calculated the grand mean to determine the level of pain-related impairment.

#### Secondary Outcomes

##### Chronic Pain

To measure the dimension chronic pain intensity at baseline and postintervention, we used the self-reported mixed scale from the German Pain Questionnaire [78] with a visual rating scale ranging from 0 (no pain) to 10 (strongest imaginable pain), measuring current, average over the last 4 weeks, and highest pain intensity. The grand mean was used for calculating the average intensity of chronic pain. In addition, the duration of chronic pain was measured with a 5-scale item from “2 to 6 months” up to “more than 5 years” [78]. We assessed the type of pain by a choice from predefined multiple-answer options: back pain, headache, facial pain, toothache, extremity pain, pelvic pain, chest pain, joint pain, tumor pain, neuralgia, whole-body pain, don’t know, other. Finally, we measured the cause of pain by the following predefined multiple-answer options: accident, surgery, inflammation, physical overload, disease, tumor, physical decline, stress, psychological strain, don’t know, other.

##### General Well-Being

We measured general well-being by the Marburger Screening for Habitual Well-being (MFHW) [79], which is a 7-item self-report questionnaire that can be scored on a 0 (not at all applicable) to 5 (totally applicable) scale. The maximum score yields 35 and means particularly good well-being. In this study, we used the grand mean of the 7 items to determine general well-being.

##### Intention of Behavior Change

To measure participants’ intention to change their behavior, we analyzed the intention to change behavior according to the Health Action Process (HAPA) [80], which differentiates three stages of change: non-intenders, intenders, and actors. Participants had to state whether they had recently been using psychological techniques for pain treatment by choosing from 1 out of 5 possible answers (No – and I do not intend to do so, No – but I’m considering it: nonintender; No – but I have the intention to do so: intender; Yes – but it’s not easy, Yes – and it’s easy: actors).

##### Working Alliance

We measured the working alliance between the participant and the TBHC SELMA with a context-adapted German version of the Working Alliance Inventory-Short Revised (WAI-SR) [81]. The WAI-SR consists of 3 dimensions targeting attachment bond (eg, “SELMA and I respect each other”), goal agreement (eg, “SELMA and I are working toward mutually agreed-upon goals”), and task agreement (eg, “I believe that the approach to working with my problem is correct”). Each subscale has 4 items with answer options ranging from never (1) to always (7). The WAI-SR was employed at the end of the intervention to assess the perceptions of the participating individuals with respect to SELMA over the course of the 8-week intervention. In addition, and owing to the importance of first impressions when interacting with a social actor (in this case SELMA), we also used the attachment bond dimension during the baseline questionnaire (ie, after SELMA has introduced herself on the first day of conversational turns).

##### Acceptance

We assessed the acceptance of the SELMA app by applying single-item measures from technology acceptance research [82,83]. In particular, single-item measures for perceived usefulness (“I think that this app is useful”), perceived ease of use (“The app is easy to use”), and perceived enjoyment (“I enjoyed using the app”), ranging from strongly disagree (1) to strongly agree (7), were employed to reduce the burden of participants. We also employed the net promoter score [84] to assess patient experience and satisfaction with the SELMA intervention (“How likely is it that you would recommend the SELMA app to people with persistent or recurring pain?”), ranging from not at all likely (0) to extremely likely (10). We assessed the duration of the intervention by the following question: “The duration of the intervention was:” with the following answer options: “too short,” “just right,” “too long.” The number of messages was assessed with the following question and answer option: “The number of chat messages was”: “too few,” just right,” “too many.” Finally, we assessed the quality of the intervention content of a chat sequence with the questions: “Was the content of the chat messages sufficient?” and the answer options “not sufficient,” “just right,” “too detailed.” The questions “What did you like most?” and “What would you like to see improved?” could be answered with free text. All of these measures were assessed at the end of the intervention.

##### Adherence

Adherence was measured by the ratio of conversations replied by participants and all conversations initiated by SELMA for the intervention and control group separately. We also assessed the relationship between the adherence ratios and study outcomes by linear regression analysis for the intervention group.

Finally, age, sex, and the highest level of education attained were assessed at baseline to describe the population of participants.

### Data Analysis

Descriptive statistics were used to summarize the characteristics of the intervention and control groups at baseline. To analyze baseline differences for demographics, we applied *t* tests and Chi-square tests. We used an independent *t* test to analyze the bond scale of the WAI-SR for differences between groups. We applied qualitative content analysis [85,86] for answers to open questions (eg, suggestions for future improvements). In particular, we explored answer text thematically with an inductive approach by defining main themes and subthemes. The frequencies of these themes were compiled in a thematic map [87]. To analyze differences between groups for pain-related impairment, pain intensity, and general well-being, we used an LMM for all participants. The model was estimated by time (T1/T2), intervention (intervene/wait), and the interaction of time and intervention as fixed factors, and participants as a random factor. We also tested the impact of the participants’ intention to change their behavior as well as the duration of pain using an LMM. The model was conducted with a restricted maximum-likelihood approach and unstructured covariance type. Missing values were replaced as missing at random with the intention-to-treat analyses. We used an LMM approach because of its ability to handle missing data and to control for type 1 error in the case of incomplete or missing data [88,89]. We calculated effect sizes for both groups (intervene/wait) on the basis of a paired sample *t* test, which compared the baseline and postintervention measures for pain-related impairment, pain intensity, and general well-being. We conducted an independent *t* test to compare adherence rates between groups, and a linear regression analysis was used for comparison of adherence rates and study outcomes. SPSS 25 (IBM Corp. Released 2017. IBM SPSS Statistics for Macintosh, Version 25.0. Armonk, NY, USA) was used for all quantitative analyses.

### Deviations from the Study Protocol

Instead of 3 variables for primary outcome (pain-related impairment, general well-being, pain intensity), we defined pain-related impairment as the primary outcome.

## Results

### Overview of Participation

**Figure 3:**
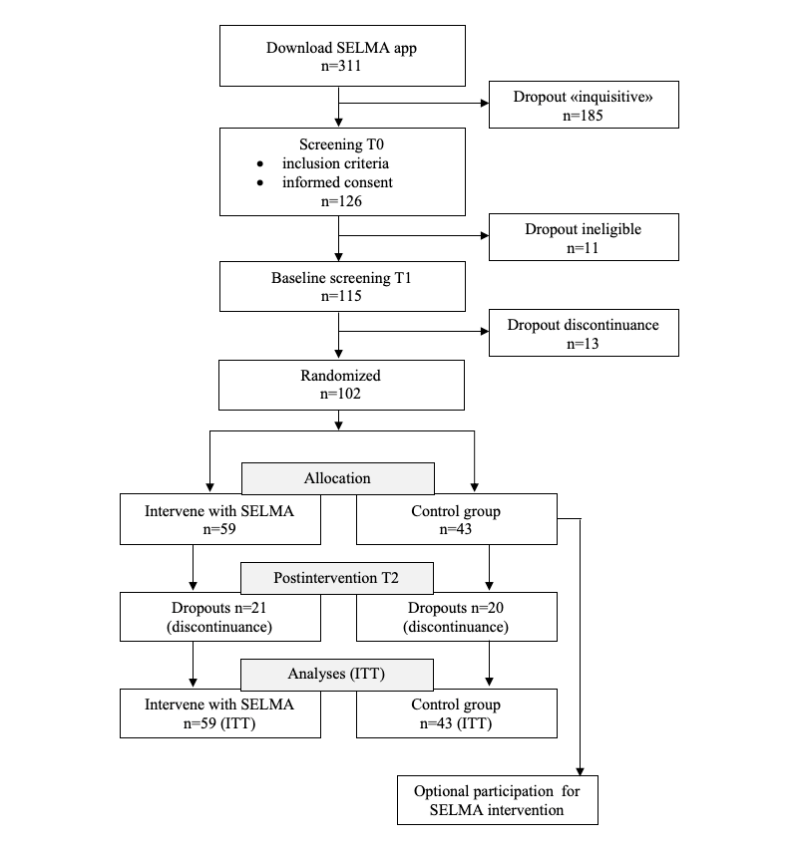
Participant flow chart. ITT: intention-to-treat.

A total of 311 downloads were made between May 5 and August 12, 2018, from which 126 individuals completed the T0 screening (inclusion criteria and informed consent), 11 of whom did not meet the criteria. The resultant sample of 115 started the T1 screening, and 13 individuals discontinued at this stage. After the onboarding process was completed, a total of 102 participants were randomized via MobileCoach automatically into either the intervention (59/102, 57.8%) or control (43/102, 42.2%) group. The participant flow chart is shown in Figure 3.

As expected, the dropout rate during the onboarding process was high. This can be explained by the fact that the app was available for anyone in App Store (Apple) and Google Play Store (Android). Some people might have downloaded the app just out of curiosity and then deleted it soon after checking in. The overall dropout rate between the baseline and follow-up screening was 21/59 (36%) for the intervention group and 20/43 (47%) for the control group. We considered only participants who did not complete the T2 screening as dropouts.

### Demographics and Baseline Scores

The demographic information and baseline scores on clinical variables for those who completed the baseline screening (N=102) are shown in Table 2. Participants were on average 43.77 (SD 12.72) years old, 82/102 (80.4%) were female, and 44/102 (43.1%) had a university degree. Overall, 55/102 (53.9%) of the participants had been suffering from pain for more than 5 years with a mean pain level of 5.61 (SD 1.66). Back pain was the most prevalent pain type in both groups (55/102, 53.9%), followed by headache (42/102, 41.2%). Means for pain-related impairment and for general well-being were 4.12 (SD 1.95) and 2.62 (SD 1.11), respectively. In terms of the intention to change behavior, 63/102 (61.0%) of the participants were classified as active, meaning they had been using psychological techniques (relaxation, mental distraction, mindfulness-based strategies, etc) recently, which were either easy or hard for them to use.

**Table 2:** Demographic and clinical variables of participants at baseline screening.

Variable			Control (n=43)	Intervention (n=59)	*P* value
Age (years), mean (SD)			44.88 (13.50)	42.97 (12.17)	.46
**Gender, n (%)**					**.07**
	Male		12 (28)	8 (14)	
	Female		31 (72)	51 (86)	
**Education, n (%)**					**.18**
	Obligatory/High school		5 (12)	3 (5)	
	Matriculation/A-Level		14 (32)	19 (32)	
	Higher vocational training		9 (21)	8 (14)	
	University		15 (35)	29 (49)	
**Duration of pain, n (%)**					**.69**
	2 to 6 months		4 (9)	3 (5)	
	6 months to 1 year		2 (5)	4 (7)	
	1 to 2 years		7 (16)	9 (15)	
	2 to 5 years		7 (16)	11 (19)	
	Over 5 years		23 (54)	32 (54)	
**Pain type, n (%)^a^**					
	Back pain		19 (44)	36 (61)	.09
	Headache		17 (40)	25 (42)	.77
	Extremities pain		17 (40)	20 (34)	.56
	Neuralgia		14 (33)	19 (32)	.97
	Joint pain		6 (14)	20 (34)	.02
	Pelvic pain		3 (7)	9 (15)	.20
	Whole-body pain		4 (9)	5 (9)	.89
	Chest pain		3 (7)	3 (5)	.69
	CRPS^b^		5 (12)	1 (2)	.04
	Facial pain		1 (2)	4 (7)	.30
	Unknown/other		1 (2)	2 (3)	.75
**Cause of pain, n (%)^a^**					
	Overstraining		12 (28)	28 (48)	.05
	Stress		9 (21)	23 (39)	.05
	Accident		13 (30)	14 (24)	.46
	Illness/migraine		9 (21)	15 (25)	.60
	Degeneration		5 (12)	19 (32)	.02
	Surgery		8 (19)	13 (22)	.68
	Inflammation		9 (21)	6 (10)	.13
	Psychogenic		6 (14)	9 (15)	.86
	Unknown/other		8 (19)	6 (10)	.22
	Gynecological		3 (7)	3 (5)	.69
	Genetic		0 (0)	3 (5)	.13
**Scale, mean (SD)**					
	Pain-related impairment (BPI^c^)		4.21 (2.00)	4.06 (1.91)	.71
	Pain intensity (DSF^d^)		5.72 (1.71)	5.52 (1.64)	.56
	General well-being (MFHW^e^)		2.65 (1.12)	2.61 (1.11)	.83
**Intention to change behavior, n (%)^f^**					**.99**
	No, and I do not plan to		6 (14)	7 (12)	
	No, but I think about it		8 (19)	10 (17)	
	No, but I have the intention to		2 (5)	6 (10)	
	Yes, but it is hard		18 (42)	26 (44)	
	Yes, and it is easy		9 (21)	10 (17)	

^a^Note that multiple selection was possible.

^b^CRPS: complex regional pain syndrome.

^c^BPI: Brief Pain Inventory.

^d^DSF: Deutscher Schmerzfragebogen (German Pain Survey).

^e^MFHW: Marbuger Fragebogen zum habituellen Wohlbefinden (Marburger Screening for Habitual Well-being).

^f^Measured by the question: Have you recently used psychological techniques to treat your pain? This includes relaxation, mindfulness, distraction, scan thoughts, etc.

### Effectiveness

Table 3 presents a comparison of the intervention and control groups from T1 to T2 for the primary outcome (pain-related impairment) as well as the secondary outcomes of pain intensity and general well-being. Means for the two groups were compared using a paired-sample *t* test. At T2, the intervention group showed significantly lower pain intensity compared to that at T1, (*t*_37_=–2.8, *P*=.009) with a moderate to large effect size (*r*=0.42) and significantly higher well-being (*t*_37_=2.41, *P*=.02) with a moderate effect size (*r*=0.37) according to Cohen [90].

**Table 3:** Results of a per-protocol paired-sample *t* test analysis comparing pre (T1) and post (T2) measures.

Outcome	Intervention (n=38)				Control (n=23)				
	T1, mean (SD)	T2, mean (SD)	*P* value	*r* ^a^		T1, mean (SD)	T2, mean (SD)	*P* value	*r*
BPI^b^	4.18 (1.81)	3.98 (2.47)	.44	0.13		4.60 (2.00)	4.19 (2.05)	.28	0.23
DSF^c^	5.85 (1.57)	5.33 (1.70)	.009	0.42		5.88 (1.75)	5.42 (1.68)	.15	0.30
MFHW^d^	2.55 (1.10)	2.93 (1.11)	.02	0.37		2.50 (1.15)	2.54 (1.10)	.76	0.01

^a^*r*: effect size.

^b^BPI: Brief Pain Inventory.

^c^DSF: Deutscher Schmerzfragebogen (German Pain Survey).

^d^MFHW: Marbuger Fragebogen zum habituellen Wohlbefinden (Marburger Screening for Habitual Well-being).

The results of LMM analyses for the entire sample including covariates are given in Table 4. At T2, participants in the intervention group did not show significantly lower levels of pain-related impairment compared to those of the control group (*t*_60_=0.42, *P*=.68).

**Table 4:** Results of the outcome intention-to-treat analysis using a linear mixed model.

Outcome		Estimate	SE	*P* value	95% CI
**Pain-related impairment (BPI^a^)**					
	Intercept	4.69	N/A^b^	N/A	N/A
	Time^c^	–0.37	0.35	.29	–1.07 to 0.33
	Group^d^	–0.13	0.38	.73	–0.89 to 0.63
	Treatment^e^	0.18	0.44	.68	–0.70 to 1.07
	HAPA^f^	0.38	0.15	.01	0.08 to 0.68
	Duration of pain	–0.12	0.16	.45	–0.44 to 0.19
**Pain intensity (DSF^g^)**					
	Intercept	5.50	N/A	N/A	N/A
	Time	–0.42	0.26	.10	–0.94 to 0.09
	Group	0.20	0.33	.55	–0.86 to 0.46
	Treatment	0.01	0.33	.97	–0.64 to 0.66
	HAPA	0.33	0.12	.01	0.08 to 0.57
	Duration of pain	0.06	0.13	.67	–0.21 to 0.32
**General well-being (MFHW^h^)**					
	Intercept	2.56	N/A	N/A	N/A
	Time	0.01	0.17	.97	–0.34 to 0.35
	Group	–0.05	0.22	.82	–0.49 to 0.49
	Treatment	0.36	0.22	.11	–0.09 to 0.80
	HAPA	–0.14	0.08	.09	–0.31 to 0.02
	Duration of pain	0.02	0.09	.80	–0.15 to 0.20

^a^BPI: Brief Pain Inventory.

^b^Not applicable.

^c^Rate of improvement for both the intervention and control groups.

^d^Intervention or control group.

^e^Represented by the group-by-time interaction.

^f^HAPA: Health Action Process (intention to change behavior).

^g^DSF: Deutscher Schmerzfragebogen (German Pain Survey).

^h^MFHW: Marbuger Fragebogen zum habituellen Wohlbefinden (Marburger Screening for Habitual Well-being).

### Intention to Change Behavior

As shown in Table 4, we found a significantly positive relation between the intention to change behavior and pain-related impairment (*t*_98_=2.54, *P*=.01) as well as pain intensity (*t*_98_=2.62, *P*=.01). The Pearson correlation coefficients between intention to change behavior and pain-related impairment (*r*=0.24; *P*=.02) as well as pain intensity (*r*=0.20; *P*=.05) revealed a significantly positive relation measured at T1. Descriptive analyses revealed that participants remained in the program by completing the T2 measure regardless of their classification as nonintenders and intenders at T1 (22/61).

### Working Alliance

Table 5 shows the mean values of the intervention group for each of the 3 dimensions of the WAI-SR (attachment bond, goal agreement, task agreement) postintervention as well as the results of the mean comparison of attachment bond between the intervention and control group pre- and postintervention. At T2, groups differed significantly in their level of attachment bond (*t*_29.6_=2.95, *P*=.005).

**Table 5:** Results of an independent t test for the subscale bond, means for subscales task and goal of the working alliance.

WAI-SR^a^	Preintervention (N=61)				Postintervention (N=61)		
	Intervention (n=38), mean (SD)	Control (n=23), mean (SD)	*P* value	Intervention (n=38), mean (SD)		Control (n=23), mean (SD)	*P* value
Total	N/A^b^	N/A	N/A	5.38		N/A	N/A
Bond	5.43 (1.27)	5.58 (1.44)	.69	5.89 (1.1)		4.51 (2.08)	.005
Task	N/A	N/A	N/A	4.95		N/A	N/A
Goal	N/A	N/A	N/A	5.3		N/A	N/A

^a^WAI-SR: Working Alliance Inventory-Short Revised (1-7 Likert scale).

^b^Not applicable.

### Acceptance

**Figure 4:**
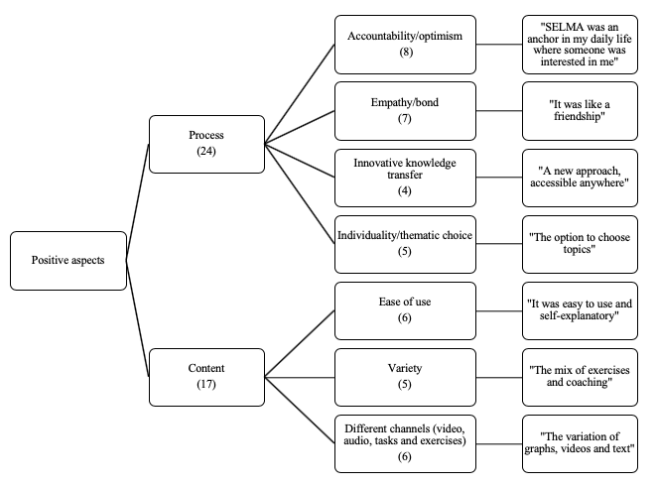
Thematic map and quotes from participants (righthand boxes) about positive aspects of the SELMA intervention. Note: numbers in brackets indicate the number of times the topic was mentioned by the participants.

**Figure 5:**
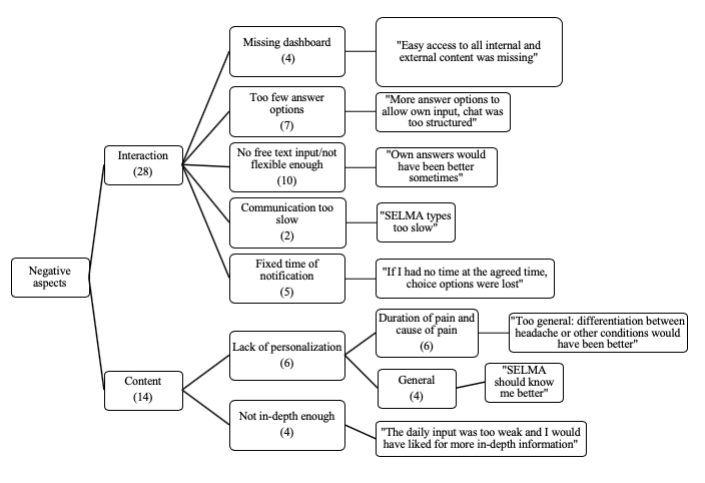
Thematic map and quotes from participants (righthand boxes) about negative aspects of the SELMA intervention. Note: Numbers in brackets indicate the number of times the topic was mentioned by the participants.

We analyzed acceptance both descriptively (Table 6) and qualitatively (Figure 4 and Figure 5), demonstrating positive feedback from the majority of users. Overall, 24/38 (63%) of the participants fully agreed or agreed that they had fun using the app, 18/38 (47%) fully agreed or agreed that the app was useful, and 31/38 (84%) agreed that it was easy to use. The duration of the program was exactly right for 19/38 (50%) of the participants. The number of messages was too short for 28/38 (74%) and their content was not sufficiently profound for 29/38 (76%) of the participants. The net promoter score was high at 16 [91].

**Table 6:** Characteristics of app acceptability for the intervention group postintervention.

Characteristic		Mean (SD) (n=38)
**The SELMA app was**		
	Enjoyable (1 to 7)	5.5 (1.45)
	Easy to use (1 to 7)	6.34 (1.15)
	Useful (1 to 7)	5.47 (1.41)
**Intervention**		
	Duration^a^	1.87 (0.70)
**Messages**		
	Number^b^	1.47 (0.76)
	Content^c^	1.37 (0.71)

^a^1=too short, 2=just right, 3=too long.

^b^1=too seldom, 2=just right, 3=too often.

^c^1=not detailed enough, 2=just right, 3=too elaborate.

Figure 4 shows a thematic map of the participants’ responses to the question “What did you like most about the app?” In the data-driven analyses, two major themes, process and content, emerged with respect to the positive aspects of the app. Regarding the process, accountability and optimism were stated most frequently (n=8), followed by empathy/bond (n=7) and ease of use and different channels (n=6). Individuality and variety were mentioned 5 times, followed by innovative knowledge transfer (n=4).

Figure 5 shows a thematic map of the participants’ responses to the question “What should be improved in the app?” *Interaction* and *content* were revealed as the main themes. Within interaction, 10 users criticized the lack of free text answers and an overly static flow of interaction. Other aspects criticized included insufficient answer options (n=7), the fixed time point of notification (n=5), a missing dashboard (n=4), and the slow communication style (n=2). The lack of personalization was further split into the subthemes *duration and type of pain* (n=6) as well as *general* (n=4). Information did not go deeply enough for some users (n=4).

### Adherence

The average adherence ratio was 71% (SD 20%) with 200 (SD 58.45) conversations initiated by SELMA in the intervention group. Participants of the control group responded with an adherence ratio of 60% (SD 27%) with 177 (SD 87.27) conversations initiated by SELMA. Multimedia Appendix 9 provides an overview of the number of conversations initiated by SELMA and responded by participants. There was no significant difference in adherence ratios between the intervention and control groups (*t*_37_=1.81, *P*=.08). Multimedia Appendix 10 shows scatter plots of the relation of adherence ratios and outcomes. Linear regression analyses showed no significant relation between adherence ratios of the intervention group and the primary outcome pain-related impairment (beta=.18, SE 1.3, *P*=.29), and the secondary outcomes pain intensity (beta=–.06, SE 0.94, *P*=.71) and general well-being (beta=.22, SE 0.77, *P*=.19).

## Discussion

### Principal Findings

To the best of our knowledge, this is the first randomized controlled trial of a fully automated, unguided, text-based conversational agent designed for patients with chronic pain. The first goal of our study was to describe the design and implementation of SELMA. We clearly demonstrated that a TBHC-based intervention can be designed and implemented for chronic pain sufferers. The second goal was to explore whether a TBHC based on CBT could help users to better self-manage their pain over a period of 8 weeks. A comparison of baseline and postintervention measures of an 8-week trial for the intervention group revealed a small effect for pain-related impairment, strong effect for pain intensity, and medium effect for general well-being. However, the control group showed similar effects. LMM analyses showed that the intervention group did not differ significantly from the control group with regard to the primary outcome, pain-related impairment.

Moreover, we found a significant relationship between participants’ intention to change behavior and both pain-related impairment as well as pain intensity. It seems plausible that people with a higher level of pain and pain-related impairment are more likely to show an increased intention to change their behavior and adopt new coping strategies in their pain self-management. Pain sufferers are more likely to change their behavior when their psychological strain increases. Additionally, participants who had no intention to change their behavior (measured at baseline) were participating and completing the intervention.

We also investigated the acceptance of the SELMA app as a secondary outcome. In general, participants enjoyed using the app, found it useful, and would recommend it to other people suffering from pain. Concerning the setting, half of the participants found the duration of the intervention to be adequate, and about the same number of users would have preferred a longer or shorter program duration. Over 70% of the users would have preferred a greater number of chat messages. Moreover, the content of messages was not extensive enough for more than 70% of the participants. Qualitative analyses revealed an insufficient degree of personalization, and many participants would have preferred to have more detailed or in-depth information about the relation of pain and behavior as well as coping strategies.

We measured adherence by assessing the ratio of conversations replied by participants and all conversations initiated by SELMA, revealing an average adherence ratio of 71% with 200 conversations initiated by SELMA; the intervention and control groups did not differ significantly. We found no significant relationship between adherence and study outcomes, although positive trends were observed. The drop-out rate was 21/59 (36%) for the intervention group and 20/43 (47%) for the control group.

Participants’ qualitative feedback indicated that many users valued the reliable and empathic aspects of interaction. This shows that they perceived a sense of interpersonal closeness with the chatbot. Results from the bond scale of the WAI-SR [25] confirmed these statements; participants in the intervention group reported significantly higher values at the follow-up measure compared to those reported at the baseline measure. By contrast, many participants wanted the option to enter more free text, feeling that the interaction was too static and not flexible. This indicates a desire of participants to interact with a chatbot in the same way they do with humans, and supports the theory of media equation, which claims that people tend to treat computers or other media as if they were real people [92] and perceive them as social actors [28].

### Comparison With Prior Work

Interventions based on a conversational agent have been recently deployed in the health sector. A recent study [93] showed that conversational agent-based interventions target neurological disorders [94-96], addictions [97,98], mental-physical wellness [99,100], nutritional metabolic disorders [101-103], and sexually transmitted disease [104]. To better compare or replicate studies, results should be reported consistently, for example according to the Consolidated Standards of Reporting Trials of electronic and mobile health applications [105]. In this study, conversational agents were used to monitor health condition, form attitudes toward health behavior, and finally intervene on cognitive or affective processes and health behavior. Scalability, personalization, consumability, asynchronicity, and anonymity were identified to be technical features of the greatest interest. Against this background, SELMA targets cognition, affect, attitude, and health behavior by using personalization. An intervention similar to SELMA [35] showed that a conversational agent in combination with medical group visits was able to reduce stress. Other related studies aim at mental-physical wellness. In one trial [99], mood was improved with a moderate effect size among users with high engagement. Another study [106] reported small to large effect sizes for improved well-being and stress among users with high engagement.

Moreover, conversational agent interventions seem to be well accepted by participants [35,101,106-108]. A meta-analysis showed that most interventions address mental health and demonstrate effectiveness [34]. Another review found that conversational agents are mainly implemented for healthy individuals rather than for those with chronic conditions [109]. Our study may contribute to closing this gap by supporting individuals suffering from chronic pain.

A more technical study categorized conversational agents regarding interaction paradigms, system architectures, and forms of dialog design [110]. This study also outlined that personalized interventions are a future challenge as are elderly populations because enhanced data are required to sustain improved support. Another study focused on conversational agents with unconstrained natural language input and found it to be an emerging field of research, but included studies that rarely evaluated efficacy or safety and were mainly quasiexperimental [111].

Furthermore, a review reported that conversational agent-based personalized interventions improved satisfaction, engagement, and dialog quality, and that outcome evaluations were often neglected [112]. These findings are promising, but because of limited evidence [113], further research is needed to identify appropriate design features for conversational agents that support pain self-management. SELMA offers personalization in various ways such as by offering the self-selection of intervention components. Qualitative analyses of SELMA revealed that participants appreciated this self-selection autonomy but wished for more personalization such as differentiation among types of pain.

Finally, there is no consensus on adherence measures for digital behavior change interventions. The ability to measure usage behavior of individuals, operationalization of intended use, and justification of intended use are deemed to be vital to measure adherence. An important aspect is to keep the goal of the app and the desired outcome in mind [114,115]; for example, assessing the time that participants spent on offline engagement with exercises, their motivation to exercise and engage with the intervention, or assessing specific elements of the intervention that were helpful to participants. Further, enjoyment of interaction with the digital coach and establishment of a working alliance might contribute to adherence, as studies have shown establishment of a working alliance between humans and health care chatbots [22,106].

### Limitations and Suggestions for Future Work

The current study has several limitations with respect to the generalizability of the results. First, as this was an exploratory pilot study, a heterogeneous sample was recruited and no follow-up data were collected to test long-term efficacy. The small sample size did not allow for further analyses such as comparing different types of pain. The heterogeneous sample also impeded establishing a definition of adequate psychoeducation. Future work should consist of larger and more homogeneous samples, along with a follow-up measure to investigate whether outcomes are persistent. This program was personalized by participants’ interest and prior knowledge. However, future programs should expand on personalization of the content by processing more pain-related personal data.

Second, the majority of users were middle-aged women. This limiting factor was also found in several other studies [14,17,116]. Even though chronic pain is more common in women than in men of Western countries [4,117], this does not fit with the distribution in this trial, and future work should motivate more men to participate in technology-driven pain management approaches. The majority of participants had a university degree, which is also a characteristic reported by other studies [7,21]. Only 6% of the users had a migration background, which does not reflect the proportion in Swiss society (37%) [118]. Perhaps immigrants have reduced access to digital interventions due to language or cultural barriers, which should be considered in future research.

Third, some participants might have inadvertently missed the timeframe set to self-select modules, resulting in SELMA selecting predefined modules. It is possible that these default modules did not correspond to the participants’ preferences and may have reduced their engagement. The default choice might have had a negative impact on the efficacy of the present study. Further research based on automated dialog systems should have flexible timepoints of communication and incorporate engagement analyses to find out more about when and for what reasons participants do not engage.

Fourth, due to technical problems, interaction with the chatbot was interrupted for some participants. The tracking of affected users was technically impossible, and some may have quit the program during this time. This factor limits an analysis of efficacy.

Fifth, in this trial, the participants had limited options to enter free text, which was criticized by many participants. Future programs should have a combination of predefined answers and free-text inputs to give users autonomy where needed. In addition, participants did not receive feedback on their progress. Individualized progress feedback is one of the most commonly used change techniques in smartphone-based health interventions [119], and helps users focus on their discrepancy awareness. Such feedback should be implemented in future programs so that participants can check to see if they are already approaching their set goals.

Finally, the control group underwent the onboarding process as well and received weekly text messages with quotations from the chatbot for ethical reasons. Because of this interaction with the chatbot, participants from this group might have started to actively challenge their pain self-management and therefore showed positive results on the primary outcomes. This might be due to the self-selecting bias that arises when individuals select themselves into a group or intervention. These individuals are particularly interested in the subject and cannot be considered to be a representative sample, as they might have different pre-existing characteristics [120]. The choice of this form of control group may have contributed to the improved levels of perceived pain-related impairment, pain intensity, and well-being.

### Conclusions

The results of the present work must be interpreted with care and the findings need to be replicated. Nonetheless, our study clearly illustrates that a TBHC-based intervention can be designed and thus offers valuable information for future program adaptions. Our findings can help to design future studies with a larger and more homogeneous sample focusing on intentional behavior change and working alliance. It is also important to further examine the usage of and adherence to digital coaching programs. Chronic pain has a high impact on personal well-being and on health costs, and innovative treatment approaches are needed. A fully automated TBHC designed to guide self-management of chronic pain could have the potential to deliver a low-threshold CBT-based coaching program for people suffering from chronic pain.

## Appendix

Multimedia Appendix 1 Overview of weekly core themes and tasks to complete.

Multimedia Appendix 2 Sympathy elements in the conversation between SELMA and a participant.

Multimedia Appendix 3 Examples of interaction with SELMA for the intervention group.

Multimedia Appendix 4 Video with various examples of interactions with SELMA.

Multimedia Appendix 5 Project website.

Multimedia Appendix 6 Flyer recruitment.

Multimedia Appendix 7 Example of an interaction with SELMA for the control group.

Multimedia Appendix 8 List of self-reported screening measures.

Multimedia Appendix 9 Adherence illustrated by number of conversations.

Multimedia Appendix 10 Scatter plots of adherence and outcomes.

Multimedia Appendix 11 CONSORT-eHEALTH checklist (V 1.6.1).

## Abbreviations

ANOVA: analysis of variance
BPI: Brief Pain Inventory
CBT: Cognitive Behavior Therapy
CRPS: complex regional pain syndrome
DRKS: German Clinical Trial Register
DSF: Deutscher Schmerzfragebogen (German Pain Survey)
HAPA: Health Action Process
KEK-ZH: Kantonale Ethikkommission Zürich (Cantonal Ethics Committee of Zurich)
LMM: Linear Mixed Model
MFHW: Marburger Fragebogen zum habituellen Wohlbefinden (Marburger Survey of Habitual Well-being)
SELMA: painSELfMAnagement chatbot
TBHC: Text-based healthcare chatbot
WAI-SR: Working Alliance Inventory-Short Revised
WHO: World Health Organization
