# Supplementary material for: A Smartphone-Based Health Care Chatbot to Promote Self-Management of Chronic Pain (SELMA): Pilot Randomized Controlled Trial
Source: JMIR Mhealth Uhealth. 2020 Apr 3;8(4):e15806. doi: 10.2196/15806 (PMC7165314; doi:10.2196/15806)
Supplement: Multimedia Appendix 1 [file mhealth_v8i4e15806_app1.pdf]

Table 1. Overview of weekly core themes and tasks to complete.

Week	Weekly core theme (source)	Brief description	Mode of communication	Task to complete
	Welcome and onboarding	Introduction, guidance through screening, randomization.	Chat-based	Provide nickname etc., informed consent, read study information and inclusion criteria, baseline screening.
1	Introduction, screening of pain-level [40]	Context of multimodal therapy in pain management. Importance of diagnostic and referral to clinics in case of problems/emergency.	Video clip, chat-based	
	Pain diary [41]	Guidance to apply pain diary over 2 weeks.	Link to PDF, chat-based	Report on pain and related situation, behavior, emotions and physical reaction.
2	Context of behavior und chronic pain [42,43]	Differentiation of acute and chronic pain.	Chat-based	
	Pain and nerves [44,45]	How an ongoing pain-stimulus and the processing of pain in the brain are related.	Link to video clip, chat-based	
	Bio-psycho-social model [42]	How chronic pain, biological, psychological factors and social environment are related.	Image, chat-based	
	Pain diary	See above		Continue to apply pain diary.
3	Pain and fear [46,55]	Vicious circle of fear and pain: How fear and negative emotions can increase pain.	Image, chat-based	
	Behavior and pain [47,55]	How trivializing, catastrophizing or social conflicts can interact with chronic pain. Protective factors.	Link to video clip, chat-based	
**4 - 8**	**Intervention modules**	**Dysfunctional cognitions, behavior, emotions**		**Self-select 3 intervention modules**
	Stress (default [a]) [43,44,46]	How stress, muscular tension and perception of pain are related. Importance of balanced level of stress.	Image, chat-based	
	Avoidance activity (default) [44,55]	Importance of physical activity. How avoiding physical activity has a negative impact on chronic pain.	Image, link to video clip, chat-based	

	Negative cognitions (default) [10,44,56]	How negative thoughts interact with pain. Vicious cycle of negative thoughts, avoidance, stress and chronic pain.	Link to video clip, image, chat-based	
	Sleep disorder [44]	How a sleeping disorder can interact with pain and vice versa. Why sleep is a protective factor against chronic pain.	Link to video clip, image, chat-based	
	Anxiety [43,44,48]	How pain and anxiety are connected with threat and how they are memorized. Vicious circle of anxiety and pain. Anxiety leads to focus on chronic pain.	Figure, chat-based	
	Perseverance [44,48]	How perseverance has immediate positive consequences but negative ones in the long run.	Link to 2 PDF-worksheets, image, chat-based	Identify level of strain and aspiration/expectations.
	Catastrophizing [44,48]	How chronic pain and catastrophizing interact.	Chat-based	
**4 - 8**	**Intervention modules**	**Coping strategies**		**Self-select 3 intervention modules**
	Relaxation (default) [44,49,50]	Meaning and relevance of relaxation. Motivation and guidance to relax.	Link to audio clip, chat-based	Relaxation, Meditation (happy place).
	Resources (default) [39,44,51]	How resources can help to relieve chronic pain. Balance model health & sickness, focusing on positive aspects.	Image, link to PDF worksheet and audio clip, chat-based	Activating resources, focusing on health.
	Mindfulness (default) [38,39,52]	Meaning of mindfulness and how to integrate into daily routine.	Link to audio clip, chat-based	Mindfulness-based exercise, relaxation.
	Physical activity[44,49,53]	How physical activity can help to reduce pain. Motivation to plan and integrate physical activity.	Link to audio clip and online-test, chat-based	Analyze suitable sports.
	Distraction [39,49]	How pain can be reduced through distraction. How attention can be controlled and changed.	Link to audio clip, image, chat-based	Meditation (Relaxation).
	Sleep [38]	Sleeping disorders and ways to cope with them.	Link to video clip and audio clip	Meditation (warmth-exercise, imaginary journey).
	Enjoyment [39]	How to learn about enjoyment despite pain.	Chat-based	Write down enjoyable things.
	Stress/strain release [44]	The need to have breaks, acceptance of strain-levels.	Link to audio clip and PDF worksheet	Mediation (bodyscan), analyze level of strain.
	Positive cognitions [39,49,51]	How to identify dysfunctional thoughts related to pain and replace them.	Link to audio clip, chat-based	Listen to story, refer to pain diary.

Acceptance [38,39,49,54]	Meaning and adoption of acceptance.	Link to audio clip	Meditation (happy place)
Medication [43]	WHO grade scheme of pain medication. Importance of correct medication. Limitation of use.	image, link to video clip, chat-based	

[a]This intervention module was selected/displayed by SELMA if a participant did not self-select a module.
