# Supplementary material for: A Smartphone-Based Health Care Chatbot to Promote Self-Management of Chronic Pain (SELMA): Pilot Randomized Controlled Trial
Source: JMIR Mhealth Uhealth. 2020 Apr 3;8(4):e15806. doi: 10.2196/15806 (PMC7165314; doi:10.2196/15806)
Supplement: Multimedia Appendix 2 [file mhealth_v8i4e15806_app2.pdf]

Sympathy and humorous elements of the conversation (translated to English from German)

Welcome back dear Mimi. How nice to have you back with me!

How nice that you, SELMA are with me too 😀

I am so pleased about your participation, and to apply mental strategies….

Me too, I am looking forward to joining you!

Dear Mimi, how do you feel today? Is it all too much for you?

I am fine, thank you 👌

More or less, SELMA 😏

You have already worked through 2 modules: very well done 💪

👍

You have gained lots of knowledge about stress and chronic pain.

🤓

How are you so far?

Very well thanks 😀

Not so well

Are you looking forward to the upcoming messages?

Sure, I do so SELMA 💃

More or less, SELMA

In any case, it is good to have you with me today to learn more basic knowledge. Soon, it will be more practical, just wait and see 😉

All right SELMA, see you later 🙋

Hopefully, you were able to learn something useful to you about fear and pain? I am look forward to guiding you thorough the next day and another topic. Yours, SELMA ☺️

Thanks SELMA and see you soon 👋

Okay, pleas have a break now – I do not want to disturb you 😴

All right, will do so staight away

Hello dear Mimi. Tell me: did you go to work by foot?

Sure, I did SELMA😉
