# Supplementary figures and images for: A Smartphone-Based Health Care Chatbot to Promote Self-Management of Chronic Pain (SELMA): Pilot Randomized Controlled Trial
Source: JMIR Mhealth Uhealth. 2020 Apr 3;8(4):e15806. doi: 10.2196/15806 (PMC7165314; doi:10.2196/15806)

## Examples of interaction with SELMA

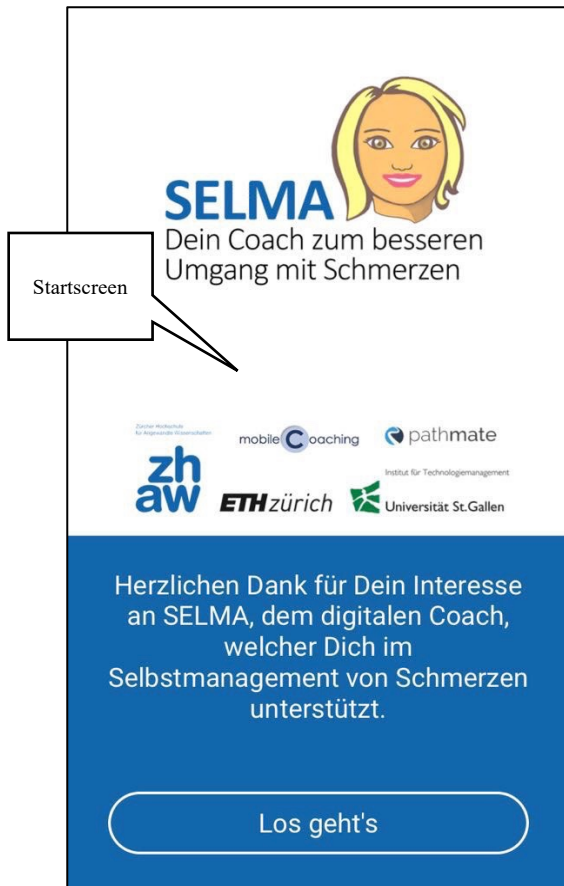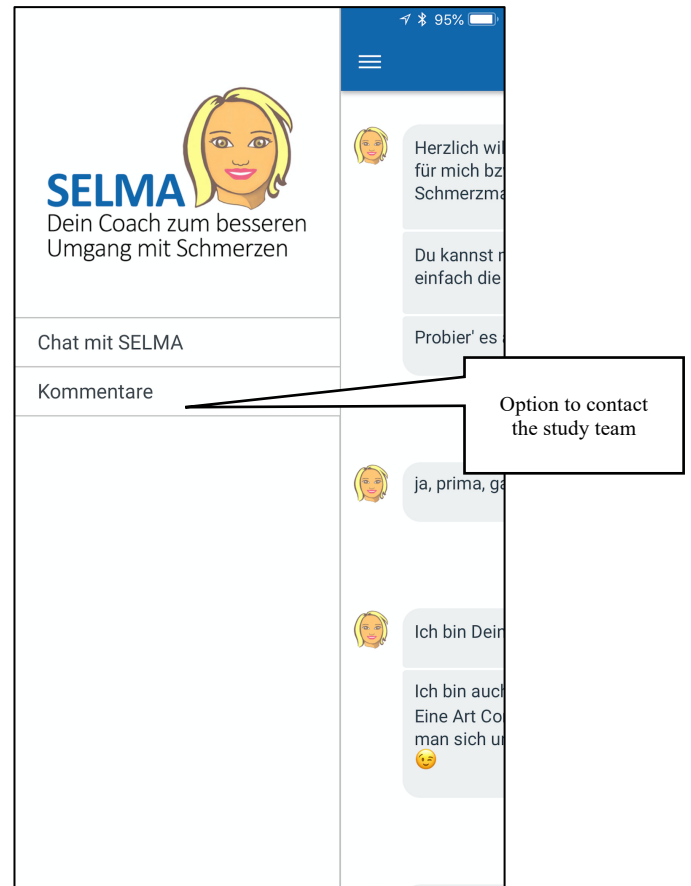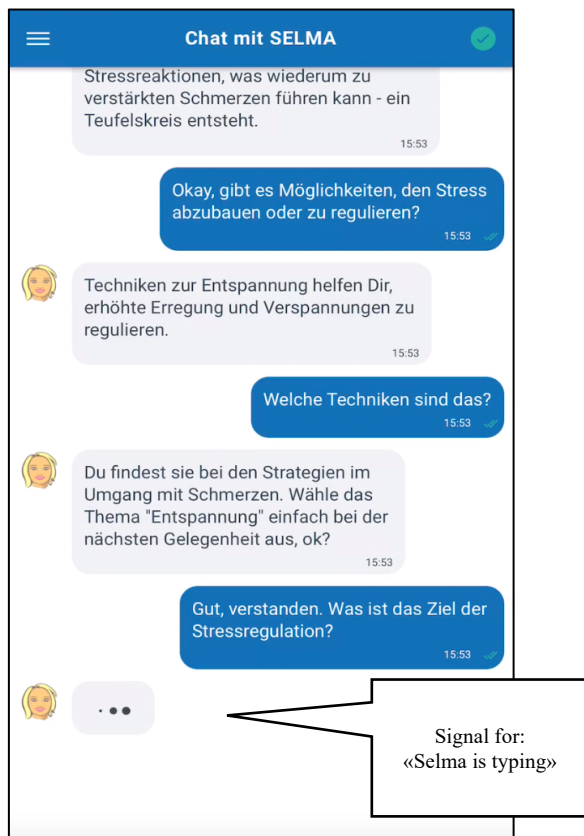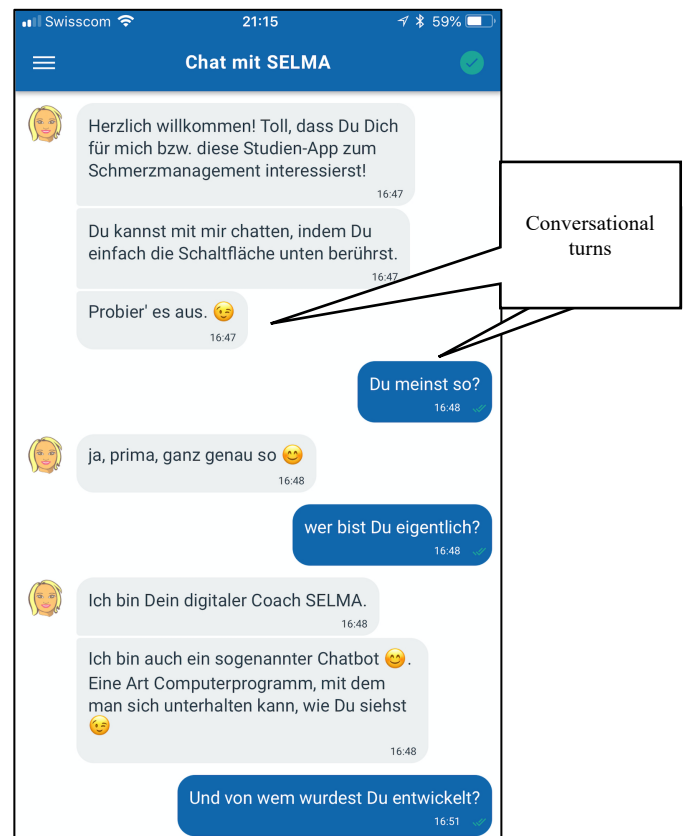

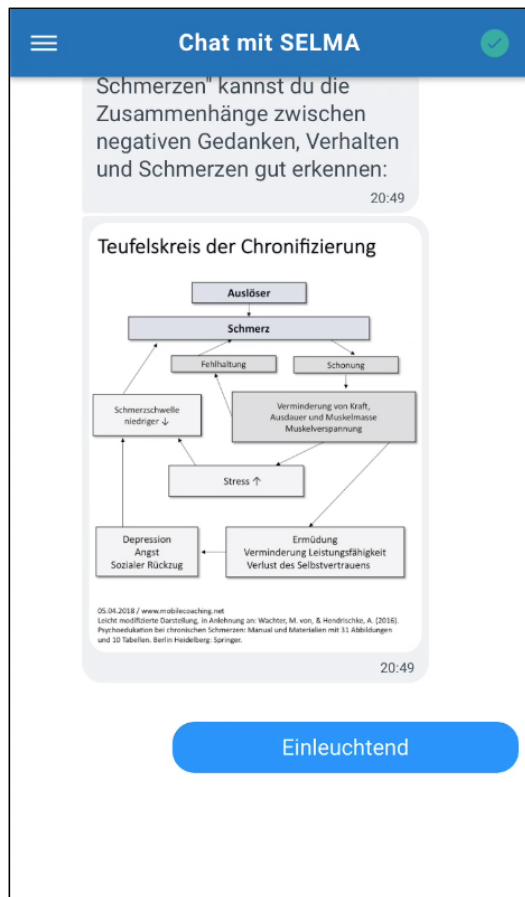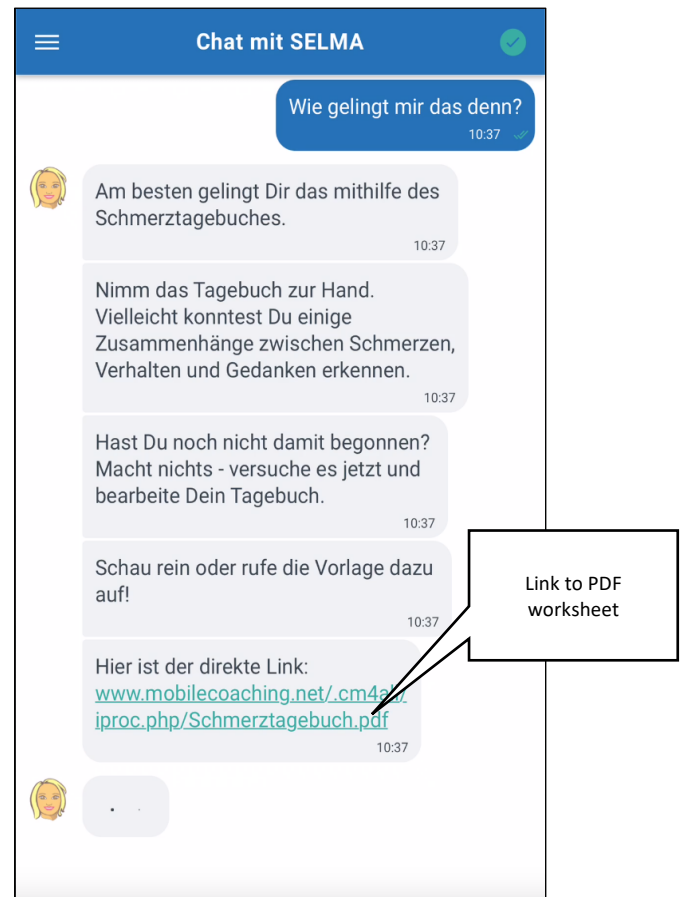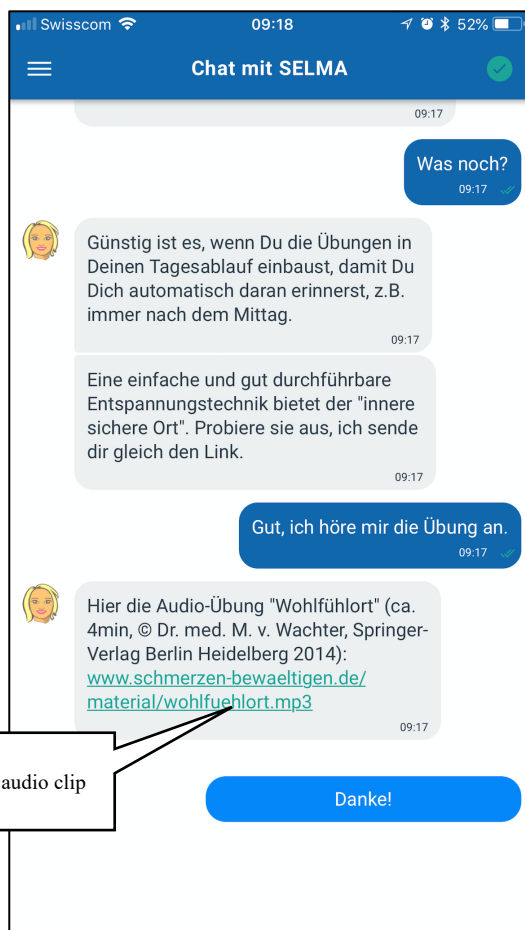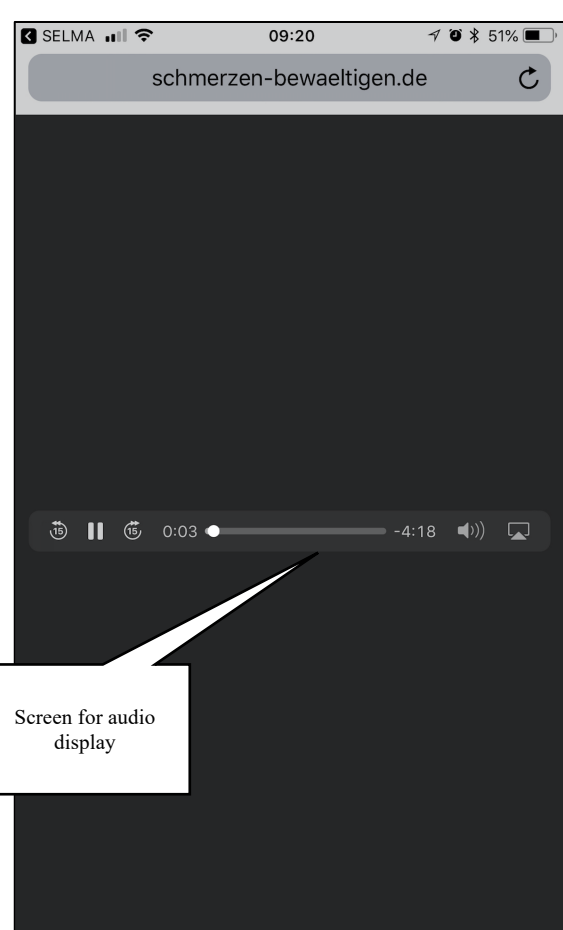

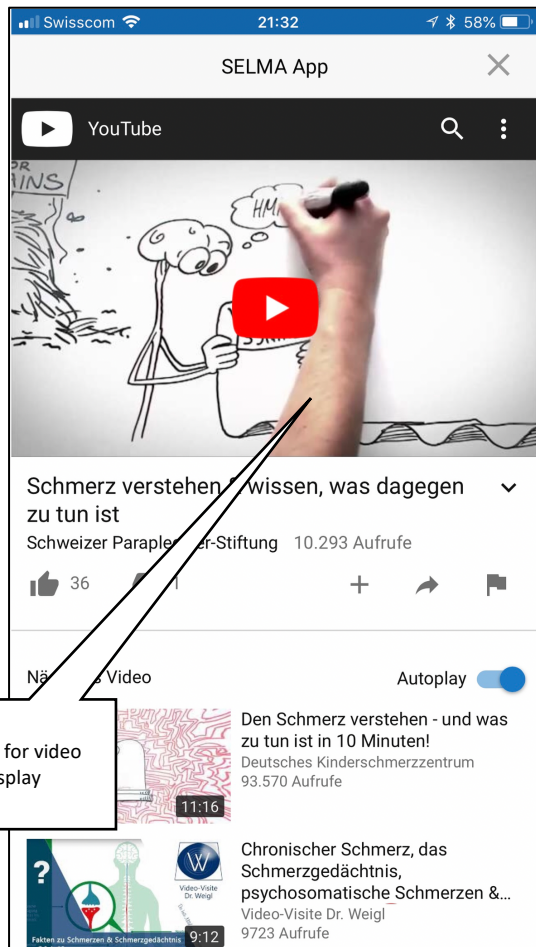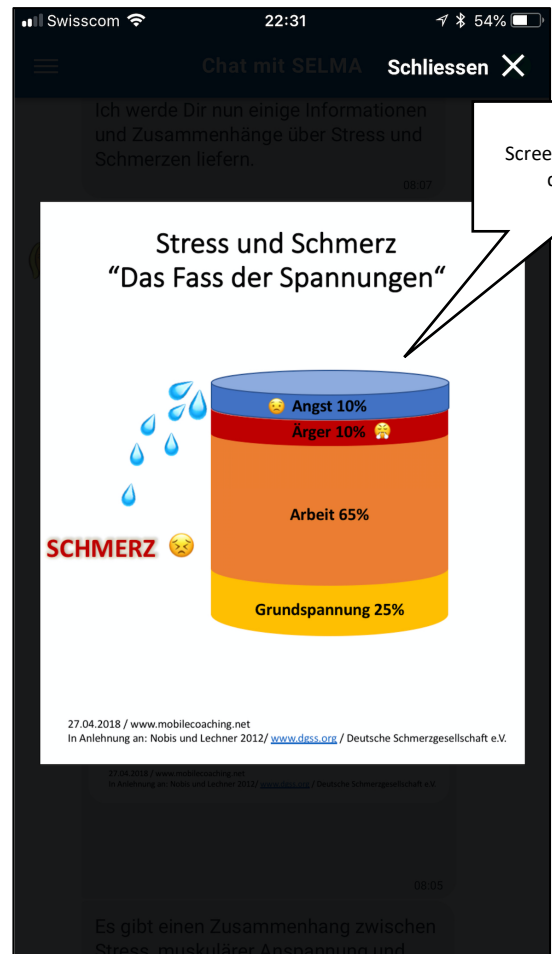

Supplement: Multimedia Appendix 3 [file mhealth_v8i4e15806_app3.pdf]

Conversations replied by participants and all conversations initiated by SELMA

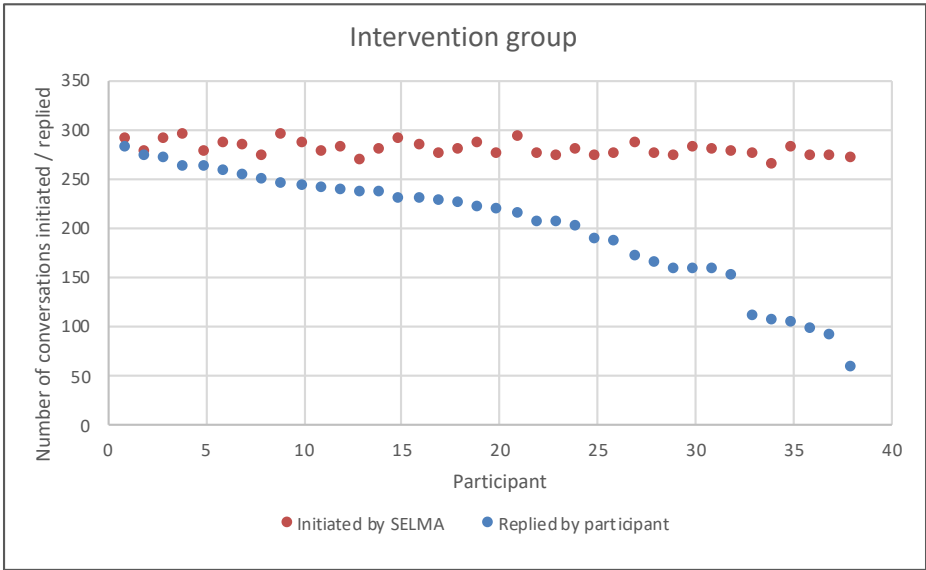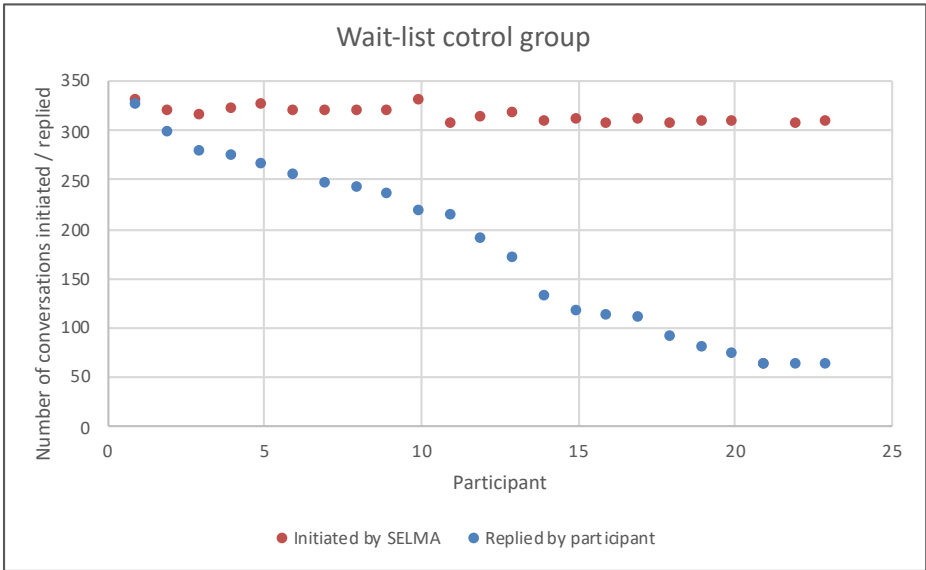

Supplement: Multimedia Appendix 9 [file mhealth_v8i4e15806_app9.pdf]
