# Supplementary material for: A Smartphone-Based Health Care Chatbot to Promote Self-Management of Chronic Pain (SELMA): Pilot Randomized Controlled Trial
Source: JMIR Mhealth Uhealth. 2020 Apr 3;8(4):e15806. doi: 10.2196/15806 (PMC7165314; doi:10.2196/15806)
Supplement: Multimedia Appendix 5 [file mhealth_v8i4e15806_app5.pdf]

Project webpage

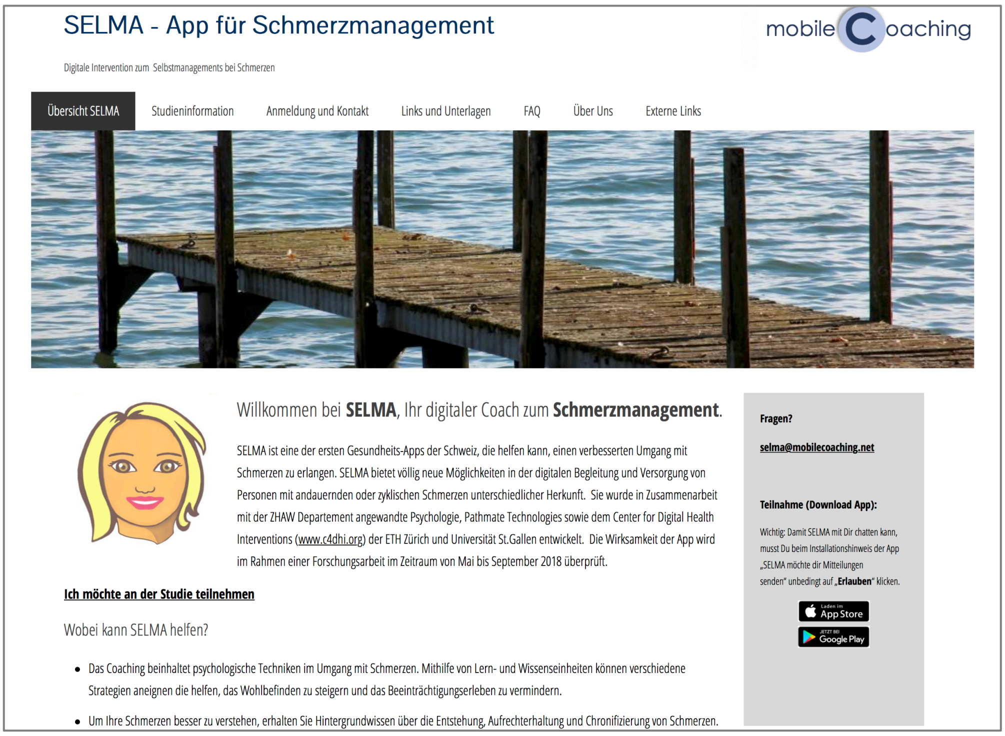

SELMA - App für Schmerzmanagement

mobile Coaching

Digitale Intervention zum Selbstmanagements bei Schmerzen

Übersicht SELMA | Studieninformation | Anmeldung und Kontakt | Links und Unterlagen | FAQ | Über Uns | Externe Links

Willkommen bei **SELMA**, Ihr digitaler Coach zum **Schmerzmanagement**.

SELMA ist eine der ersten Gesundheits-Apps der Schweiz, die helfen kann, einen verbesserten Umgang mit Schmerzen zu erlangen. SELMA bietet völlig neue Möglichkeiten in der digitalen Begleitung und Versorgung von Personen mit andauernden oder zyklischen Schmerzen unterschiedlicher Herkunft. Sie wurde in Zusammenarbeit mit der ZHAW Departement angewandte Psychologie, Pathmate Technologies sowie dem Center for Digital Health Interventions (www.c4dhi.org) der ETH Zürich und Universität St.Gallen entwickelt. Die Wirksamkeit der App wird im Rahmen einer Forschungsarbeit im Zeitraum von Mai bis September 2018 überprüft.

Ich möchte an der Studie teilnehmen

Wobei kann SELMA helfen?

- Das Coaching beinhaltet psychologische Techniken im Umgang mit Schmerzen. Mithilfe von Lern- und Wissenseinheiten können verschiedene Strategien aneignen die helfen, das Wohlbefinden zu steigern und das Beeinträchtigungserleben zu vermindern.
- Um Ihre Schmerzen besser zu verstehen, erhalten Sie Hintergrundwissen über die Entstehung, Aufrechterhaltung und Chronifizierung von Schmerzen.

Fragen?

selma@mobilecoaching.net

Teilnahme (Download App):

Wichtig: Damit SELMA mit Dir chatten kann, musst Du beim Installationshinweis der App „SELMA möchte dir Mitteilungen senden" unbedingt auf „**Erlauben**" klicken.

Laden im App Store

JETZT BEI Google Play

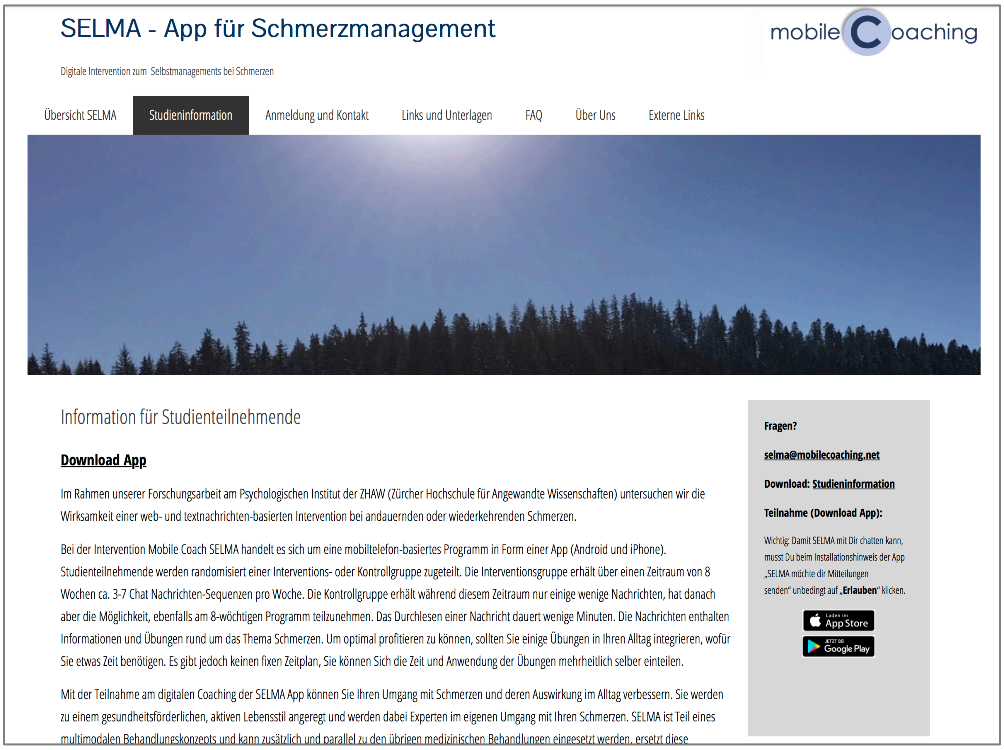

SELMA - App für Schmerzmanagement

mobile Coaching

Digitale Intervention zum Selbstmanagements bei Schmerzen

Übersicht SELMA | Studieninformation | Anmeldung und Kontakt | Links und Unterlagen | FAQ | Über Uns | Externe Links

Information für Studienteilnehmende

Download App

Im Rahmen unserer Forschungsarbeit am Psychologischen Institut der ZHAW (Zürcher Hochschule für Angewandte Wissenschaften) untersuchen wir die Wirksamkeit einer web- und textnachrichten-basierten Intervention bei andauernden oder wiederkehrenden Schmerzen.

Bei der Intervention Mobile Coach SELMA handelt es sich um eine mobiltelefon-basiertes Programm in Form einer App (Android und iPhone). Studienteilnehmende werden randomisiert einer Interventions- oder Kontrollgruppe zugeteilt. Die Interventionsgruppe erhält über einen Zeitraum von 8 Wochen ca. 3-7 Chat Nachrichten-Sequenzen pro Woche. Die Kontrollgruppe erhält während diesem Zeitraum nur einige wenige Nachrichten, hat danach aber die Möglichkeit, ebenfalls am 8-wöchtigen Programm teilzunehmen. Das Durchlesen einer Nachricht dauert wenige Minuten. Die Nachrichten enthalten Informationen und Übungen rund um das Thema Schmerzen. Um optimal profitieren zu können, sollten Sie einige Übungen in Ihren Alltag integrieren, wofür Sie etwas Zeit benötigen. Es gibt jedoch keinen fixen Zeitplan, Sie können Sich die Zeit und Anwendung der Übungen mehrheitlich selber einteilen.

Mit der Teilnahme am digitalen Coaching der SELMA App können Sie Ihren Umgang mit Schmerzen und deren Auswirkung im Alltag verbessern. Sie werden zu einem gesundheitsförderlichen, aktiven Lebensstil angeregt und werden dabei Experten im eigenen Umgang mit Ihren Schmerzen. SELMA ist Teil eines multimodalen Behandlungskonzepts und kann zusätzlich und parallel zu den übrigen medizinischen Behandlungen eingesetzt werden, ersetzt diese

Fragen?

selma@mobilecoaching.net

Download: Studieninformation

Teilnahme (Download App):

Wichtig: Damit SELMA mit Dir chatten kann, musst Du beim Installationshinweis der App „SELMA möchte dir Mitteilungen senden" unbedingt auf „**Erlauben**" klicken.

Laden im App Store

JETZT BEI Google Play
