# Supplementary material for: A Smartphone-Based Health Care Chatbot to Promote Self-Management of Chronic Pain (SELMA): Pilot Randomized Controlled Trial
Source: JMIR Mhealth Uhealth. 2020 Apr 3;8(4):e15806. doi: 10.2196/15806 (PMC7165314; doi:10.2196/15806)
Supplement: Multimedia Appendix 6 [file mhealth_v8i4e15806_app6.pdf]

Winter/Frühjar 2018
Studienorganisator:
Psychologisches Institut
ZHAW Angewandte Psychologie
Pfingstweidstrasse 96
8005 Zürich

Zürcher Hochschule
für Angewandte Wissenschaften
zh aw Angewandte Psychologie

Sponsor: Prof. Hansjörg Künzli, ZHAW Angewandte Psychologie
Studienleiterinnen: Sandra Hauser-Ulrich und Danielle Meier-Peterhans, BSc ZFH in Angewandter Psychologie / Stud. MSc

---

Information

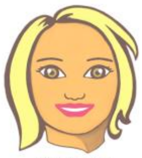

SELMA

SELMA – App für Schmerzmanagement

SELMA ist eine der ersten Gesundheits-App, die Dir helfen kann, einen verbesserten Umgang mit Deinen Schmerzen zu erlangen. SELMA bietet völlig neue Möglichkeiten in der digitalen Begleitung und Versorgung von Personen mit andauernden oder zyklischen Schmerzen unterschiedlicher Herkunft.

SELMA wurde in Zusammenarbeit mit der ZHAW Departement angewandte Psychologie und dem Center for Digital Health Interventions von der ETH Zürich und Universität St. Gallen entwickelt. Die Wirksamkeit der App wird im Rahmen einer Forschungsarbeit überprüft.

Die Anwendung des Coachings macht Dich zum eigenen Experte im Umgang mit Deinen Schmerzen und vermittelt Dir psychologische Techniken und Hintergrundwissen. Du kannst den Fokus auf bestimmte Themen richten und entscheiden, in welchen Bereichen Du Dir Wissen aneignen möchtest.

Das individuelle Coaching kannst Du ganz bequem von zu Hause aus durchführen. Du bekommst über einen Zeitraum von 8 Wochen alle ein bis zwei Tage einige Chat Nachrichten von SELMA. Das Durchlesen einer Nachricht dauert wenige Minuten. Um optimal profitieren zu können, solltest Du einige Übungen in Deinen Alltag integrieren, wofür Du etwas Zeit benötigst.

Die Zugangsdaten zum Coaching sowie weitere Informationen findest Du auf der Website
http://www.mobilecoaching.net/

Kontaktpersonen

Bei Fragen, Unsicherheiten oder Anregungen, kannst Du Dich an eine dieser Kontaktpersonen wenden.

Sandra Hauser-Ulrich und Danielle Meier-Peterhans
BSc ZFH in Angewandter Psychologie / Stud. MSc

E-Mail selma@mobilecoaching.net

SCAN MICH!

Herzlichen Dank für Dein Interesse!

Winter/Frühjar 2018
Studienorganisator:
Psychologisches Institut
ZHAW Angewandte Psychologie
Pfingstweidstrasse 96
8005 Zürich

Sponsor: Prof. Hansjörg Künzli, ZHAW Angewandte Psychologie
Studienleiterinnen: Sandra Hauser-Ulrich und Danielle Meier-Peterhans, BSc ZFH in Angewandter Psychologie / Stud. MSc

Information

SELMA – App für Schmerzmanagement

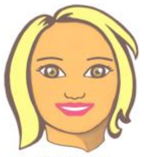
SELMA

SELMA ist eine der ersten Gesundheits-App, die Dir hilft, einen verbesserten Umgang mit Deinen Schmerzen zu erlangen. SELMA bietet völlig neue Möglichkeiten in der digitalen Begleitung und Versorgung von Personen mit andauernden oder zyklischen Schmerzen unterschiedlicher Herkunft.

SELMA wurde in Zusammenarbeit mit der ZHAW Departement angewandte Psychologie und dem Center for Digital Health Interventions von der ETH Zürich und Universität St. Gallen entwickelt. Die Wirksamkeit der App wird im Rahmen einer Forschungsarbeit überprüft.

Die Anwendung des Coachings macht Dich zum eigenen Experte im Umgang mit Deinen Schmerzen und vermittelt Dir psychologische Techniken und Hintergrundwissen. Du kannst den Fokus auf bestimmte Themen richten und entscheiden, in welchen Bereichen Du Dir Wissen aneignen möchtest.

Das individuelle Coaching kannst Du ganz bequem von zu Hause aus durchführen. Du bekommst über einen Zeitraum von 8 Wochen alle ein bis zwei Tage einige Chat Nachrichten von SELMA. Das Durchlesen einer Nachricht dauert wenige Minuten. Um optimal profitieren zu können, solltest Du einige Übungen in Deinen Alltag integrieren, wofür Du etwas Zeit benötigst.

Die Zugangsdaten zum Coaching sowie weitere Informationen findest Du auf der Website
http://www.mobilecoaching.net/

Kontaktpersonen

Bei Fragen, Unsicherheiten oder Anregungen, kannst Du Dich an eine dieser Kontaktpersonen wenden.

Sandra Hauser-Ulrich und Danielle Meier-Peterhans
BSc ZFH in Angewandter Psychologie / Stud. MSc

E-Mail selma@mobilecoaching.net

SCAN MICH!

Herzlichen Dank für Dein Interesse!
