# Supplementary material for: A Smartphone-Based Health Care Chatbot to Promote Self-Management of Chronic Pain (SELMA): Pilot Randomized Controlled Trial
Source: JMIR Mhealth Uhealth. 2020 Apr 3;8(4):e15806. doi: 10.2196/15806 (PMC7165314; doi:10.2196/15806)
Supplement: Multimedia Appendix 8 [file mhealth_v8i4e15806_app8.pdf]

List of self-reported screening measures

Time of measure	Outcome	Basis
Baseline	Sociodemographic	German Pain-Screening (DSF), Deutsche Schmerzgesellschaft, IASP, 2015
Baseline and post-intervention	Pain intensity	German Pain-Screening (DSF), Deutsche Schmerzgesellschaft, IASP, 2015
Baseline	Pain duration	German Pain-Screening (DSF), Deutsche Schmerzgesellschaft, IASP, 2015
Baseline	Type and genesis of pain	German Pain-Screening (DSF), Deutsche Schmerzgesellschaft, IASP, 2015
Baseline and post-intervention	Pain related impairment	Brief Pain Inventory (BPI), Subscale impairment, Radbruch et al., 1999
Baseline and post-intervention	General well-being	Marburger screening for habitual well-being (MFHW), Herda, Scharfenstein & Basler, 1998
Baseline	Intention of behavior change	Health Action Process Approach (HAPA), Schwarzer, 1992
Baseline and post-intervention	Working Alliance (bond scale)	Working Alliance Inventory – short revised (WAI-SR) German version, Wilmers et al, 2008
Post-intervention, intervention group only	Working Alliance (task and goal scale)	Working Alliance Inventory – short revised (WAI-SR) German version, Wilmers et al, 2008
Post-intervention, intervention group only	Acceptance	The Net Promoter Score, Krol, de Boer, Delnoij & Rademakers, 2015; User acceptance of hedonic information systems, van der Heijden, 2004
