# Supplementary material for: A Smartphone-Based Health Care Chatbot to Promote Self-Management of Chronic Pain (SELMA): Pilot Randomized Controlled Trial
Source: JMIR Mhealth Uhealth. 2020 Apr 3;8(4):e15806. doi: 10.2196/15806 (PMC7165314; doi:10.2196/15806)

Scatter Plot adherence (ratio conversations responded) with impairment, general well-being and pain intensity

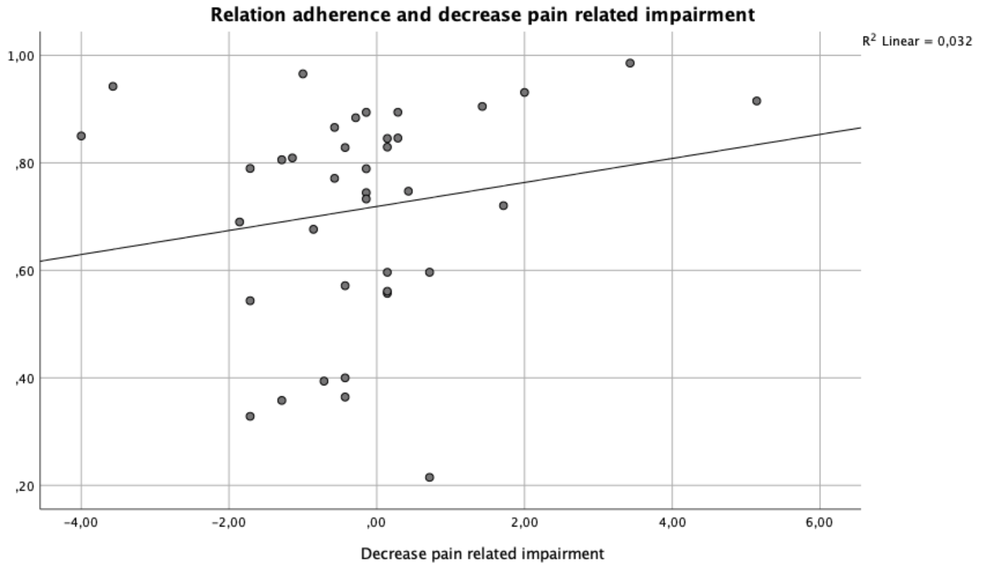

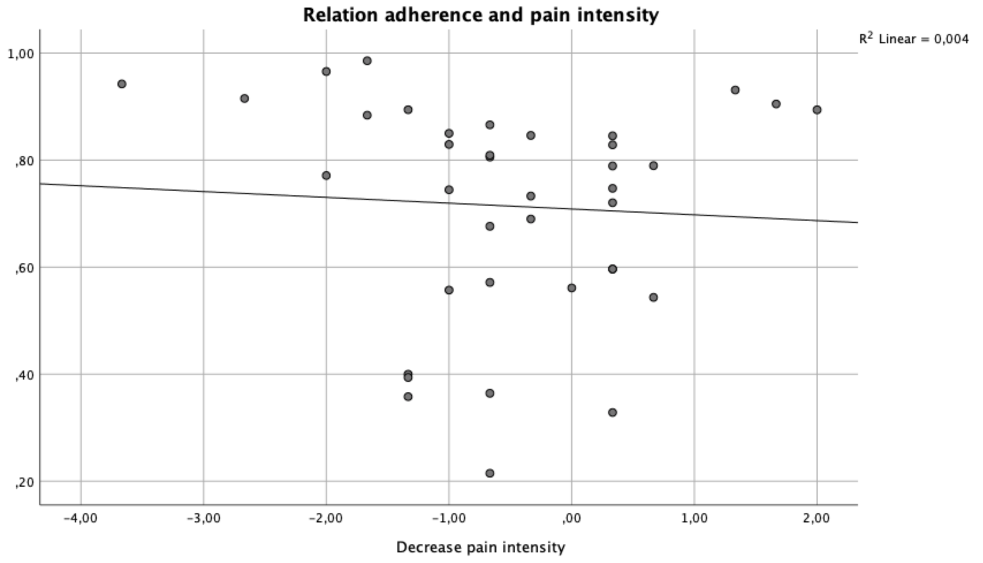

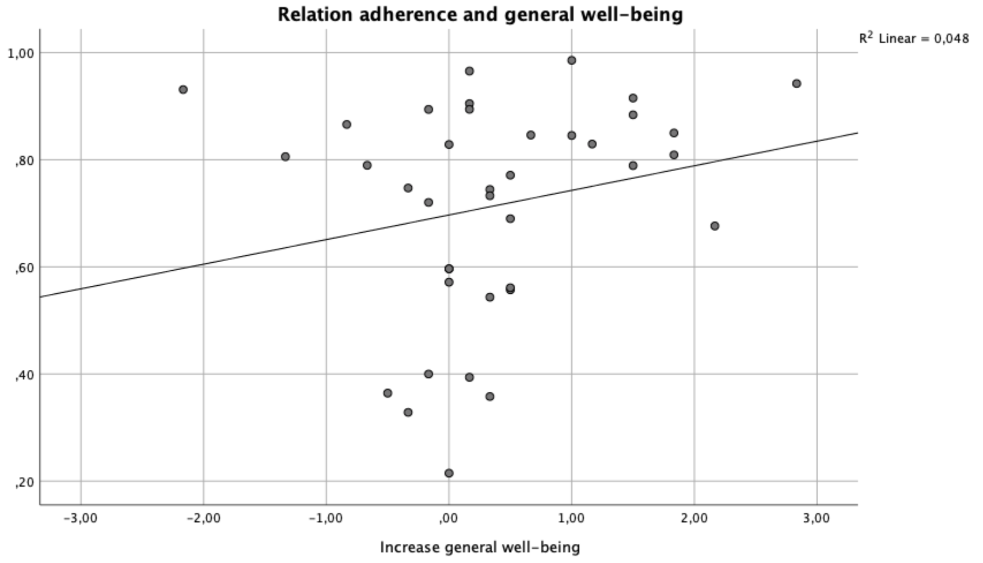

Supplement: Multimedia Appendix 10 [file mhealth_v8i4e15806_app10.pdf]
